# Accessing Ancestral Origin and Diversity Evolution by Net Divergence of an Ongoing Domestication Mediterranean Olive Tree Variety

**DOI:** 10.3389/fpls.2021.688214

**Published:** 2021-06-24

**Authors:** Hélia Sales, Zlatko Šatović, Mara Lisa Alves, Pedro Fevereiro, João Nunes, Maria Carlota Vaz Patto

**Affiliations:** ^1^Instituto de Tecnologia Química e Biológica António Xavier, Universidade Nova de Lisboa, Oeiras, Portugal; ^2^Centre Bio R&D Unit, Association BLC3 – Technology and Innovation Campus, Lagares, Oliveira do Hospital, Portugal; ^3^Faculty of Agriculture, University of Zagreb, Zagreb, Croatia; ^4^Centre of Excellence for Biodiversity and Molecular Plant Breeding (CoE CroP-BioDiv), Zagreb, Croatia; ^5^InnovPlantProtect - Collaborative Laboratory, Estrada de Gil Vaz, Elvas, Portugal

**Keywords:** *Olea europaea*, genetic diversity, genetic erosion, SSR, ancient and centennial trees, minimum spanning tree

## Abstract

*Olea europaea* ‘Galega vulgar’ variety is a blend of West and Central Mediterranean germplasm with cultivated-wild admixture characteristics. ‘Galega vulgar’ is known for its high rusticity and superior-quality olive oil, being the main Portuguese variety with high impact for bioeconomy. Nevertheless, it has been replaced by higher-yielding and more adapted to intensive production foreign varieties. To clarify the potential ancestral origin, genetic diversity evolution, and existing genetic relationships within the national heritage of ‘Galega vulgar’, 595 trees, belonging to ancient and centenary age groups and prospected among ten traditional production regions, were characterized using 14 SSR markers after variety validation by endocarp measurements. Ninety-five distinguishable genets were identified, revealing the presence of a reasonable amount of intra-genetic and morphological variability. A minimum spanning tree, depicting the complete genealogy of all identified genets, represented the ‘Galega vulgar’ intra-varietal diversity, with 94% of the trees showing only a two-allele difference from the most frequent genet (C001). Strong correlations between the number of differentiating alleles from C001, the clonal size, and their net divergence suggested an ancestral monoclonal origin of the ‘Galega vulgar’, with the most frequent genet identified as the most likely origin of all the genets and phenotypic diversification occurring through somatic mutations. Genetic erosion was detected through the loss of some allele combinations across time. This work highlights the need to recover the lost diversity in this traditional olive variety by including ancient private genets (associated with potential adaptation traits) in future breeding programs and investing in the protection of these valuable resources *in situ* by safeguarding the defined region of origin and dispersion of ‘Galega vulgar’. Furthermore, this approach proved useful on a highly diverse olive variety and thus applicable to other diverse varieties due either to their intermediate nature between different gene pools or to the presence of a mixture of cultivated and wild traits (as is the case of ‘Galega vulgar’).

## Introduction

*Olea europaea* L. subsp. *europaea* var. *europaea* (Green, 2002), commonly known as olive tree, is one of the main agroecological symbols of the Mediterranean Basin. It has been extensively cultivated for thousands of years in this region, primarily to produce olive oil and/ or table olives (Breton et al., 2012). Nuclear and plastid DNA data has shown that the main wild progenitor of the cultivated olive is the wild Mediterranean olive, also known as oleaster (*O*. *europaea* subsp. *europaea* var. *sylvestris*) (Besnard et al., 2018). According to archaeological and genetic studies, the domestication of the cultivated olive seems to have occurred after the emergence of major human civilizations in the Middle East ~6,000 years ago, in the Neolithic (Besnard et al., 2013b, 2018). New lines of evidence indicate the existence of multiple centers of diversity for cultivated olive trees, but it remains unclear whether the centers of diversity resulted from one or multiple local domestication events (Besnard et al., 2018). The olive tree germplasm is abundant in genetic diversity either in cultivated or in wild forms (Breton et al., 2006; Baldoni and Belaj, 2009; Belaj et al., 2010; Julca et al., 2020). In the cultivated forms, the long life span and vegetative propagation of these trees across centuries have resulted in the occurrence of “hidden” smaller genetic differences within varieties, i.e., intra-variety variations that only rarely are expressed as morphological differences (Belaj et al., 2004; Díez et al., 2011; Trujillo et al., 2014). The characterization of olive variability, including intra-varietal variability and the relationships between cultivated and wild olives, is of utmost importance for different areas. Knowledge gained can promote an efficient conservation of existing genetic resources, an effective broadening of the genetic basis of breeding programs, the development of molecular-based selection breeding tools or oil traceability tools, and an effective nursery management. In addition, it might also contribute for the clarification of the varieties' ancestral origin (Gemas et al., 2004; Lopes et al., 2004; Muzzalupo et al., 2010, 2014; Strikić et al., 2011; Ipek et al., 2012; Atienza et al., 2013; Trujillo et al., 2014; Díez et al., 2016; Sion et al., 2019; Li et al., 2020).

In 2019/2020, Portugal ranked seventh in the worldwide production of olive oil (~4.39%), and it is the fourth-largest olive producer in Europe, after Spain, Greece, and Italy (IOC, 2020^1^) Hence, the olive sector assumes key importance in national agricultural policy and economy, having generated in the last 3 years a higher turnover (around €620 million) compared with 2010 and 2012 (GPP, 2019^2^) In Portugal, as in the other traditional olive-growing countries, there are a number of olive varieties (Cordeiro et al., 2014), with the national production mostly concentrated on 22 varieties (Leitão et al., 1986). In particular, the ‘Galega vulgar’ variety, also called ‘Galega’, is the main variety in Portugal (Cordeiro et al., 2008), associated with five of the six national Protected Designations of Origin (PDO) regions (Gouveia, 2002). This variety is characterized by alternate bearing and high rusticity (Cordeiro et al., 2014), and stands out for the excellent quality of its olive oil (Cordeiro et al., 2008). Its resistance to drought is also recognized, despite its moderate productivity, which is partly due to sensitivity to different pests (Cordeiro et al., 2008, 2014). Despite all these interesting traits, the ‘Galega vulgar’ variety has been recently replaced in the national olive groves by higher-yielding foreign varieties, more adapted to intensive production systems (Linos et al., 2014). Illustrating this replacement, data from 2007/2008 showed ‘Galega vulgar’ accounting for 80% of the olive trees in the country (Cordeiro et al., 2008), while more recently, only 60% of all olive trees in Portuguese olive groves corresponds to ‘Galega vulgar’ variety (Arias-Calderón et al., 2017). This mounting replacement may have already led to the loss of alleles or of combinations of alleles, in a clear case of genetic erosion (Maxted and Guarino, 2006; Brown and Hodgkin, 2015). There is an urgent need to better characterize the currently available diversity to efficiently conserve and promote the use of this national genetic richness. Only in this way will it be possible to counteract the potential ‘Galega vulgar’ genetic erosion, improving yields and adaptation, and restoring the cultivation of this high-quality variety. The few studies performed on this variety, namely by assessing its genetic variability (Gemas et al., 2004; Lopes et al., 2004; Cordeiro et al., 2008; Figueiredo et al., 2013), were all limited in the number of genotypes analyzed, with no clear comparison between what is presently under production in newer orchards and a more ancestral but still existing diversity. In this context, a proper characterization of the state of the ‘Galega vulgar’ intra-variability is still missing.

Nowadays, molecular markers are fundamental tools used in the characterization and understanding of diversity, due to their independence from environmental effects (Hammer et al., 2003). In the diversity analysis of olive trees, microsatellites, also called simple sequence repeats (SSRs), are the molecular markers of choice because of transfer ability, high polymorphic, co-dominance, and their relatively simple interpretation (Belaj et al., 2003). This type of molecular marker has been successfully used for olive tree variety identification, genetic diversity studies, and to evaluate the relationships between different varieties (Sarri et al., 2006; Bracci et al., 2009; Ercisli et al., 2011; di Rienzo et al., 2018; Lazović et al., 2018; El Bakkali et al., 2019; Dervishi et al., 2021). Due to the wide use of SSRs in olive trees, Baldoni et al. (2009) published a set of recommended microsatellite markers and protocols for olive genotyping, along with a list of reference varieties suitable for the establishment of a worldwide database of genotypes.

In the present study, 595 trees of different ages were characterized using SSR markers, after the validation of the ‘Galega vulgar’ variety by endocarp measurements, to clarify the overall genetic diversity evolution, potential ancestral origin, and genetic relationships within the national heritage of ‘Galega vulgar’. Centennial individuals, which represent the olive trees still in production at the Portuguese orchards, were combined with ancient individuals (estimated 400–2,000 years old), to trace back any eventual loss of allelic diversity that might have occurred across time. This study will allow us to track and recover the genetic diversity still existing in the country but that might be already lost in the present ‘Galega vulgar’ orchards; broaden the available genetic basis for breeding purposes; and unravel the ancestral origin of this variety. All these efforts will contribute to the efficient conservation of this variety and to promote its use.

## Materials and Methods

### Plant Material

**Figure 1:**
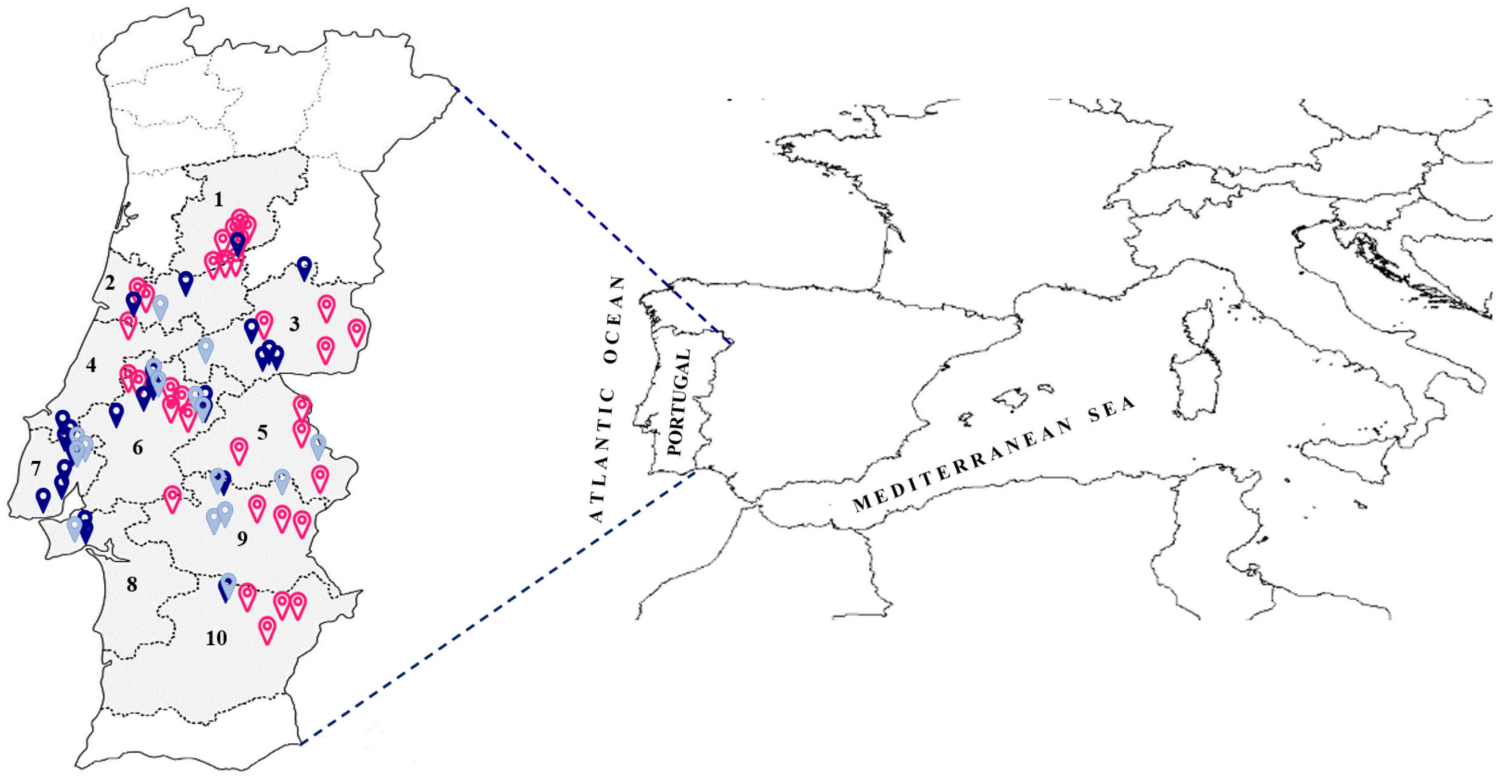
Map of Portugal with the location of prospected orchards and isolated sites from traditional regions of olive production. Empty pink dots—orchards with centenary ‘Galega vulgar’; solid gray dots—orchards with ancient ‘Galega vulgar’; solid blue dots—isolated ancient ‘Galega vulgar’; numbers—capital city of the ten prospected geographical districts (1–Viseu; 2–Coimbra; 3–Castelo Branco; 4–Leiria; 5–Portalegre; 6–Santarém; 7–Lisboa; 8–Setúbal; 9–Évora; 10–Beja).

To assess the diversity of the ‘Galega vulgar’ variety present in the Portuguese orchards or kept on isolated trees, a collection was established with individual trees prospected from different traditional regions of olive production. Prospections were performed taking into consideration the age of the olive trees provided by farmers or conservation sites (centennial trees still under production, between 80 and 100 years old, and ancient trees, present both in orchards or in isolated sites, about 400–2,000 years old). Within each orchard, the prospected olive trees (about ten trees/orchard) were randomly selected and tagged with a unique identifier, and the tree global position system (GPS) coordinates recorded. The same procedure was used for isolated individuals. This resulted in 629 prospected trees, 356 of which were centennial and 273 ancient, from ten geographical districts (Figure 1). A complete list of the trees prospected, along with their “passport” information, endocarp profile and calculated age in years (according to Michelakis, 2002; Koniditsiotis, 2020), is available in Supplementary Table 1 and the agroecological characterization of the ten geographical districts is available in Supplementary Table 2.

For each tree, leaf and fruit samples were collected in two different seasons: during autumn, 50 fruits *per* tree were collected to perform endocarp validation of the ‘Galega vulgar’ variety; and during spring, ten young leaves *per* tree were collected for DNA isolation and genetic diversity characterization. The fruits were kept on ice at harvest and then stored at 4 ± 2°C until variety validation, while leaves were kept on ice when collected and then frozen in liquid nitrogen and stored at −80 ± 2°C until genomic DNA extraction.

Representative sample of each ‘Galega vulgar’, ‘Arbequina’, and ‘Picual’ varieties from the World Olive Germplasm Bank (WOGB) were also included in the SSR analysis.

### Morphological Endocarp Characterization

**Figure 2:**
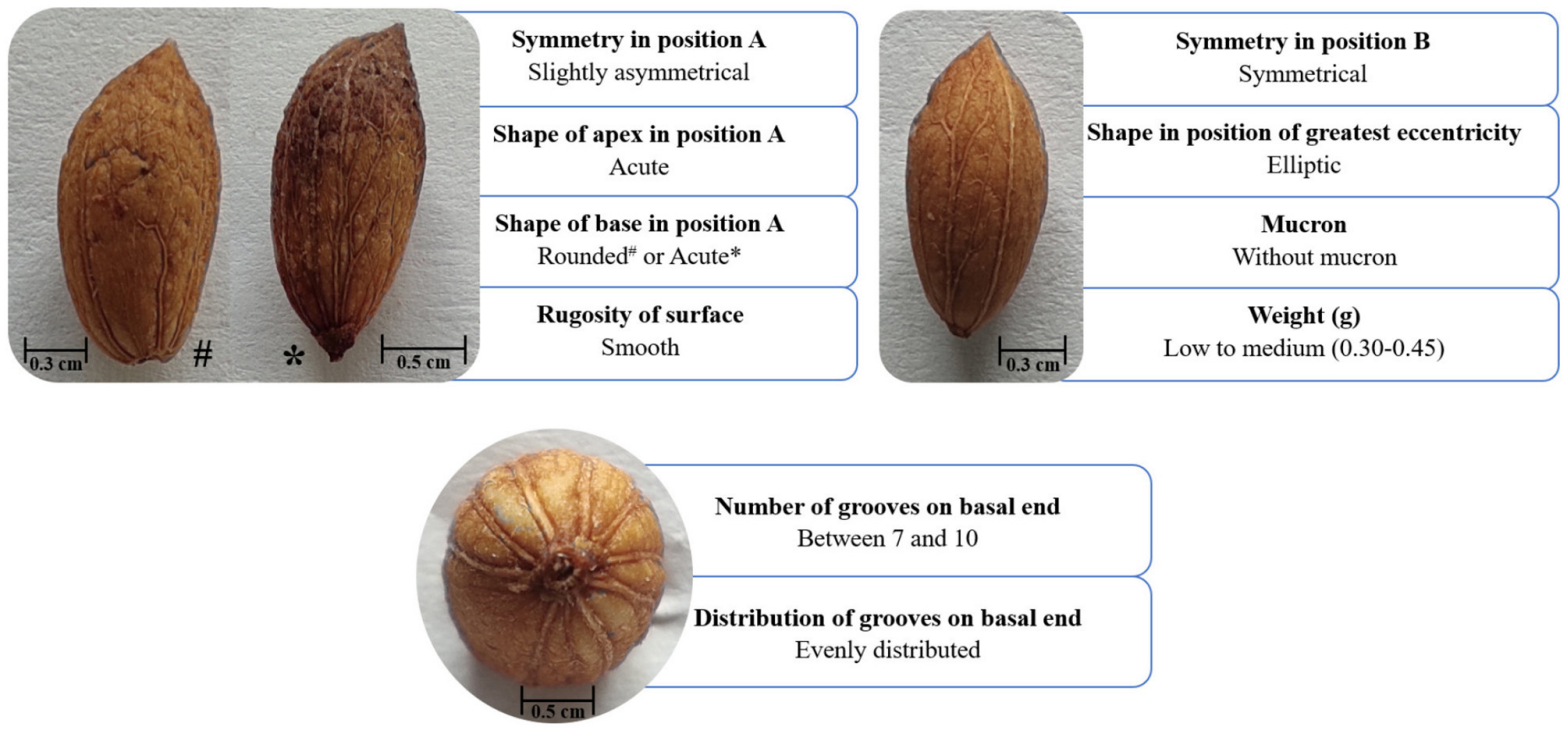
Endocarp characterization of ‘Galega vulgar’ variety, using a pomological scheme of nine qualitative and one quantitative morphological parameters (Leitão et al., 1986; Cordeiro et al., 2010; CVO, 2012^3^). The qualitative parameters (symmetry in position A, shape of apex in position A, shape of the base in position A, rugosity of surface, symmetry in position B, shape in the position of greatest eccentricity, mucron, number of grooves on basal end and distribution of grooves on basal end) were visually assessed using traits states, while the quantitative parameter (endocarp weight) was measured according to CPVO (2012^3^).

The validation of the ‘Galega vulgar’ variety was performed through a morphological description limited to qualitative and quantitative traits, using a pomological scheme (CPVO, 2012^3^) Specimens' vouchers of the ‘Galega vulgar’ endocarps and leaves are deposited at Instituto de Tecnologia Química e Biológica António Xavier and Association BLC3, Technology and Innovation Campus institutions. Fifty endocarps, randomly sampled *per* each of the 629 prospected trees, were characterized using nine different qualitative parameters and one quantitative parameter: shape in the position of greatest eccentricity, symmetry in position A, symmetry in position B, number of grooves on basal end, distribution of grooves on basal end, shape of apex in position A, mucron, shape of the base in position A, rugosity of surface, and weight (Figure 2). The qualitative parameters were visually assessed using traits states, while the quantitative parameter “endocarp weight” was measured according to CPVO (2012^3^). Trees were validated as belonging to the ‘Galega vulgar’ variety if all the evaluated parameters corresponded to the endocarp descriptions referenced in Leitão et al. (1986) and Cordeiro et al. (2010). These two criteria are still widely used but show differences concerning the shape of the base in position A [acute or rounded in Leitão et al. (1986) vs. rounded in Cordeiro et al. (2010)], and weight [<0.30 g in Leitão et al. (1986), vs. 0.30–0.45 g in Cordeiro et al. (2010)]. Therefore, a combination of both criteria was applied. To validate the analyzed trees as ‘Galega vulgar’, endocarp parameters needed to fall within the following four different profiles: endocarp with rounded base shape in position A and 0.30–0.45 g weight, rounded base shape in position A and weight <0.30 g, acute base shape in position A and 0.30–0.45 g weight, and acute base shape in position A and weight <0.30 g. Figure 2 shows the observed variation in ‘Galega vulgar’ variety's endocarp parameters.

### Molecular Characterization

#### DNA Extraction and Microsatellites Analysis

DNA was extracted from the sampled young leaves using the CTAB procedure developed by Doyle and Doyle (1987). The quality of the extracted DNA was assessed by electrophoresis on 0.80% SeaKem^®^ LE Agarose gel (Lonza, Rockland, USA), stained with SYBR^®^ Safe (Invitrogen, Eugene, USA), and visualized using a GEL-DOC1000 System (Bio-Rad, Hercules, USA). Quantification and extra quality evaluation were performed using a Nanodrop spectrophotometer, Nanodrop ND-2000C (Thermo Scientific, USA).

The SSR analysis consisted of 14 SSRs, selected from the consensus list of the best olive SSRs (Baldoni et al., 2009): ssrOeUA-DCA03, ssrOeUA-DCA05, ssrOeUA-DCA09, ssrOeUA-DCA10, ssrOeUA-DCA11, ssrOeUA-DCA14, ssrOeUA-DCA16, ssrOeUA-DCA18, EMO-90, GAPU71B, GAPU89, GAPU101, GAPU103A, and UDO99-043. The SSR loci were amplified using the method for fluorescent labeling of PCR fragments (Schuelke, 2000). Accordingly, a M13 tail was added to the 5'-end of the forward primers, allowing the annealing of the universal M13(-21) primer labeled with IRDye fluorescence and the visualization of the amplified fragments. Each PCR reaction was carried out in a total volume of 10 μL, containing 10 ng of template DNA, 1 x PCR buffer (Promega, Madison, USA), 1.50 mM of MgCl2, 0.20 mM of each dNTP, 0.04 μM of M13(-21) tagged forward primer, 0.16 μM of IRD700 or IRD800 M13(-21), 0.16 μM of reverse primer and 0.20 units of Taq DNA polymerase (Promega, Madison, USA). The amplification reactions consisted of a denaturing step of 94°C for 5 min, followed by 30 cycles of 94°C for 30 s, 56°C for 45 s, 72°C for 45 s and 8 cycles of 94°C for 30 s, 53°C for 45 s, 72°C for 45 s, with a final step of 72°C for 10 min. After the amplification, 1 μL from each reaction product was blended with 25 μL of formamide-loading buffer (98% formamide, 10 mM EDTA pH = 8 and 0.10% Bromo Phenol Blue). The mixture was vortexed, heated for 5 min at 95°C, and then cooled on ice. 0.50 to 0.70 μl of each sample was loaded on a 6.50% denaturing polyacrylamide gel (KBPlus Gel Matrix, LI-COR), using an LI-COR 4300 DNA Analyzer (Lincoln, NE, USA). DNA from two ‘Galega vulgar’ trees (randomly chosen from our initial data set), were used as reference samples, to account for any variation that could occur between PCR reactions and electrophoresis runs.

Allele sizes (in base pairs) of PCR products were estimated using SagaGT software from LI-COR and data compiled in a matrix for further analysis. All alleles were double scored and manually reviewed by two independent researchers to prevent scoring errors. Samples with small differences in allele sizes were additionally checked by re-amplifications to exclude possible genotyping and allele sizing errors. The ‘Galega vulgar’, ‘Arbequina’, and ‘Picual’ representative samples from the WOGB were also included in the allele sizes estimation.

#### Microsatellite Diversity Analysis

The overall diversity detected by the SSR markers was assessed calculating the total number of alleles (N_a_) and genotypes (N_g_) *per* locus as well as the Polymorphic Information Content (PIC) (Botstein et al., 1980) of each microsatellite marker, using PowerMarker v3.23 software (Liu and Muse, 2005).

#### Identification of Genets

The number of distinct multi-locus genotypes (i.e., genets) in the set of ‘Galega vulgar’ tree samples was identified using GenClone 2.0 (Arnaud-Haond and Belkhir, 2007). According to Harper (1997), individuals that develop by vegetative propagation of the same parental plant are referred to as ramets, while the entire set consisting of multiple ramets sharing the same multi-locus genotypes is referred to as a genet. Therefore, from now we will use these terms in the context of ‘Galega vulgar’. Using GenClone 2.0 software, the Pareto distribution of multi-locus genotypes (the inverse cumulated frequency of distinct multi-locus genotypes including *x* ramets) was plotted on a log-log scale. The Pareto's parameter β was calculated by regression from the Pareto distribution as −1 multiplied by the regression slope (Arnaud-Haond et al., 2007). As described by Arnaud-Haond et al. (2007), a high evenness with genets that all have comparable sizes results in a steep slope (i.e., a high β value), whereas the result of a skewed distribution with few large and many small genets is a shallow slope (i.e., a low β-value).

To represent the genetic relationships among the identified ‘Galega vulgar’ genets, a principal coordinate analysis based on the pairwise distance matrix was performed. The pairwise genetic distances were calculated based on proportion-of-shared-alleles distance [D_psa_; Bowcock et al. (1994) as implemented in MICROSAT (Minch et al., 1997)]. A principal coordinate analysis (PCoA) was carried out using NTSYSpc v. 2.11a software (Rohlf, 1998).

The correlation between the ‘Galega vulgar’ genets and the ‘Galega vulgar’ endocarps' profiles for the studied trees was calculated using the R software ver. 3.4.2. (R Core Team, 2017).

#### Intra-Varietal Diversity Analysis

After the validation of the variety with the endocarp analysis, the presence of intra-variability in ‘Galega vulgar’ was considered when allelic differences were detected among the analyzed trees, and according to Trujillo et al. (2014), corresponding to molecular variants of ‘Galega vulgar’.

Intra-varietal diversity among all validated ‘Galega vulgar’ samples was analyzed overall but also considering different groupings: (1) germplasm groups [centennial trees (<100 years old) vs. ancient trees (>400 years old)], (2) age groups [centennial C1 trees (<100 years old), ancient A1 (ranging from 400 to 600 years), ancient A2 (ranging from 600 to 900 years), ancient A3 (more than 1,000 years old)], (3) groups according to endocarp profile (E1–E4), (4) geographical groups (10 regions of origin), and (5) groups according to tree location (orchard vs. isolated tree).

Intra-varietal allelic diversity was evaluated, by calculating the average number of alleles *per* locus (*N*_*av*_), the allelic richness (*N*_*ar*_) as a measure of the number of alleles *per* locus independent of sample size, and the number of private alleles (*N*_*pr*_), using FSTAT v. 2.9.3.2 software (Goudet, 2002).

Intra-varietal clonal diversity was assessed by calculating the number of multi-locus genotypes (*N*_*c*_), clonal richness (*N*_*cr*_), and genotypic richness [*R*; Dorken and Eckert (2001)], using GenClone 2.0 (Arnaud-Haond and Belkhir, 2007). Simpson's complement index [D^*^; Simpson (1949), Arnaud-Haond et al. (2007)], describing the probability of encountering distinct genets when randomly taking two trees of a given variety, and Simpson's evenness index [V; Hurlbert (1971), Fager (1972), Arnaud-Haond et al. (2007)], describing clonal equitability [closely associated with diversity as it is a means of assessing evenness among relative abundances (Valbuena et al., 2012)] were also calculated using the same software. As the number of multi-locus genotypes (*N*_*c*_) is dependent on sample size, the clonal richness (*N*_*cr*_) was calculated according with the modified formula proposed by El Mousadik and Petit (1996) for calculating allelic richness (*N*_*ar*_), based on the rarefaction method of Hurlbert (1971):

$$\begin{array}{l} Nar=\sum_{c=1}^{C}\left[1-\frac{\left(\frac{N-Nc}{n}\right)}{\left(\frac{N}{n}\right)}\right] \end{array}$$

where: N–sample size, *n*–subsample size, *N*_*c*_–number of samples belonging to the multi-locus genotype *c*, C–total number of multi-locus genotypes. Therefore, this parameter was used as a measure of the number of multi-locus genotypes *per* group independent of sample size.

Intra-varietal genetic diversity was analyzed after identifying the tree samples that shared an identical genet and removing the duplicates from the data set. GENEPOP 4.0 (Raymond and Rousset, 1995) was used to calculate the observed (*H*_*O*_) and expected (*H*_*E*_) heterozygosity within each group.

The differences in *N*_*ar*_, *H*_*O*_, and *H*_*E*_ between the groups were tested across microsatellite markers by the analysis of variance using PROC GLM in SAS v. 9.3 (SAS, 2011), followed by Tukey's HDS test when more than two groups were compared.

The partitioning of total genetic variation of genets between and within groups was analyzed by AMOVA (Excoffier et al., 1992) using Arlequin ver. 3.5.2.1 (Excoffier et al., 2005). The variance components were tested using 10,000 permutations.

To check if some genets were particularly divergent from the rest, the net divergence of each genet was calculated by summing up the number of different alleles of a genet to all the other genets included in the analysis. To analyze the relationship between the amount of divergence and the clonal size, several correlations were computed and tested between (A) the net divergence of the genets and the logarithm of the number of ramets or clonal size, (B) the number of different alleles of each genet to the most frequent genet (C001) and the net divergence of the genet, and (C) the number of different alleles of each genet to the most frequent genet (C001) and the number of ramets in each genet (SAS, 2011).

The minimum spanning tree based on the matrix of genetic distances computed as the number of different alleles among genets was constructed using a custom script in Python version 3.6 (available on request) and the tree was visualized using Graphviz (https://www.graphviz.org/). The tree was rooted using the most frequent genet (C001) as outgroup.

## Results

### ‘Galega Vulgar’ Variety Validation

According to the endocarp morphological analysis (based on Figure 2), of the 629 prospected olive trees, 595 were within the boundaries of the four endocarp profile types described for ‘Galega vulgar’ variety (Table 1) (complete data set available online at FigShare repository). The most common profile, found in 350 trees (~58.82% ‘Galega vulgar’ trees), was the one with a “rounded base shape in position A” combined with a “medium weight” (between 0.30 and 0.45 g). All the four profiles were found among the prospected ‘Galega vulgar’ trees. The ‘non-Galega vulgar’ samples (34 trees) were excluded from further analysis.

**Table 1:** Morphological characterization of endocarps and the endocarp profiles found in the 595 ‘Galega vulgar’ trees.

**No. of trees**	**Endocarp profile**^**a**^	**Morphological characteristics of the endocarp**									
		**Shape in position of greatest eccentricity^**b**^**	**Symmetry (position A)^**c**^**	**Symmetry (position B)^**d**^**	**N^**°**^. of grooves^**e**^**	**Distribution of grooves^**f**^**	**Apex shape (position A)^**g**^**	**Mucron^**h**^**	**Base shape (position A)^**i**^**	**Surface^**j**^**	**Weight^**k**^**
350	1	EP	SA	S	M	R	A	A	R	S	M
59	2	EP	SA	S	M	R	A	A	R	S	L
162	3	EP	SA	S	M	R	A	A	A	S	M
24	4	EP	SA	S	M	R	A	A	A	S	L

a *Olive endocarp profile: codes from 1 to 4 were assigned based on different morphological profiles*.

b *Shape in the position of greatest eccentricity: EP, elliptical*.

c *Symmetry (position A): SA, slightly asymmetric*.

d *Symmetry (position B): S, symmetric*.

e *Number of grooves: M, medium (7–10)*.

f *Distribution of grooves: R, regular distribution*.

g *Apex shape (position A): A, acute*.

h *Mucron: A, absent*.

i *Base shape (position A): A, acute; R, rounded*.

j *Surface: S, smooth*.

k *Weight: low, L (<0.30 g); medium, M (0.30–0.45 g)*.

### Microsatellite Diversity in the ‘Galega Vulgar’ Variety

Using 14 microsatellite markers, a total of 77 different alleles was identified in the entire collection of 595 validated ‘Galega vulgar’ genotypes (Table 2) (complete data set available online at FigShare repository). The number of alleles *per locus* varied from 1 (EMO-90) to 9 (GAPU103A), with an average of 5.50. The most informative SSRs were ssrOeUA-DCA11 and GAPU103A, showing a PIC value of 0.61 and 0.57, respectively (Table 2). As the EMO-90 marker showed to be monomorphic for all the ‘Galega vulgar’ samples and the GAPU71B did not differentiate among samples (all the samples had the same heterozygous genotype), they were both excluded from the subsequent analysis. Regarding the samples from WOGB, the ‘Galega vulgar’ representative sample presented the same genotype as the one found in the majority of the collected ‘Galega vulgar’ samples (Table 2). ‘Arbequina’ and ‘Picual’ differed from the ‘Galega vulgar’ most frequent genotype in all the SSRs used, with the exception of the ssrOeUA-DCA14 (‘Arbequina’ and ‘Picual’) and the EMO-90 (‘Picual’).

**Table 2:** Allelic diversity of 14 microsatellite loci in a set of 595 ‘Galega vulgar’ tree samples.

**No**.	**Locus**	**Size range**	**N_**a**_**	**N_**g**_**	**PIC**	**Most frequent genotype**
M01	ssrOeUA-DCA03	236–253	7	7	0.44	236/250
M02	ssrOeUA-DCA05	204–216	3	2	0.39	204/212
M03	ssrOeUA-DCA09	179–197	8	11	0.48	191/193
M04	ssrOeUA-DCA10	148–164	6	6	0.17	158/158
M05	ssrOeUA-DCA11	127–186	8	7	0.61	127/179
M06	ssrOeUA-DCA14	178–188	4	4	0.03	188/188
M07	ssrOeUA-DCA16	157–180	8	9	0.39	176/176
M08	ssrOeUA-DCA18	171–185	4	3	0.39	171/179
M09	EMO-90	185–185	1	1	0.00	185/185
M10	GAPU71B	119–125	2	1	0.37	119/125
M11	GAPU89	160–210	8	7	0.48	160/204
M12	GAPU101	184–205	6	4	0.41	185/201
M13	GAPU103A	141–192	9	8	0.57	141/186
M14	UDO99-043	173–179	3	3	0.03	173/173
Mean			5.50		0.34	
Total			77			

### ‘Galega Vulgar’ Variety Genet Identification

#### Number of Multi-Locus Genotypes or Genets

**Figure 3:**
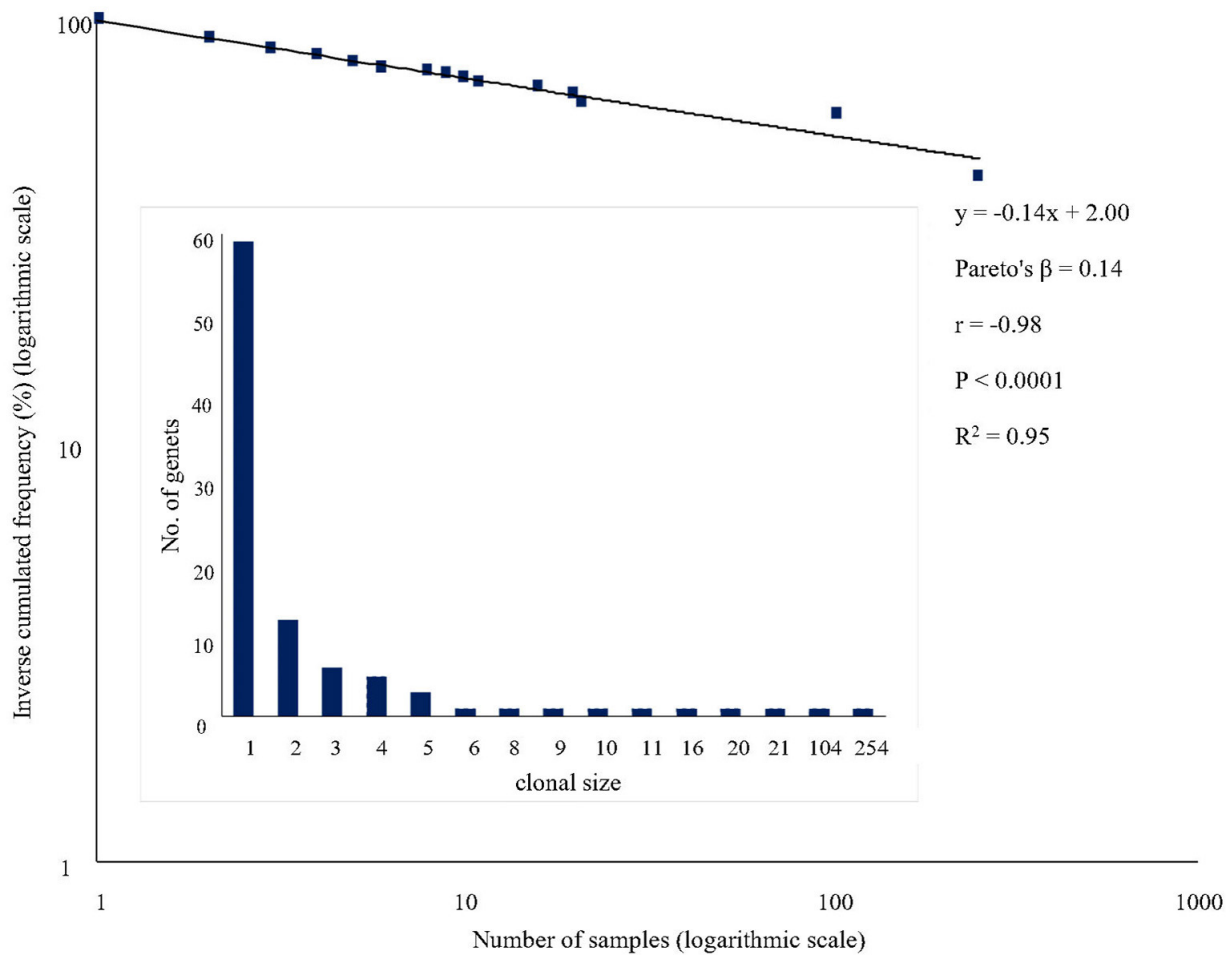
Pareto's plot of ‘Galega vulgar’ samples: Linear regression equation, Pareto's distribution coefficient (Pareto's β), correlation coefficient (r), significance of the correlation (P) and coefficient of determination (R^2^) are given for ‘Galega vulgar’ variety. The histogram shows the distribution of the number of tree samples among genets.

A total of 95 different multi-locus genotypes (or genets) was identified in the panel of the 595 trees analyzed. The distribution of genets according to the number of genetically identical samples (clonal size) was highly skewed, with two large genets and many small ones resulting in a shallow regression line of Pareto's plot (Figure 3). The largest genet (genet C001) was found in 254 out of 595 genotypes (42.69%), followed by C002 found in 104 genotypes (17.48%). Thus, the largest two genets represented more than 60% of the ‘Galega vulgar’ trees studied and from the remaining 93 distinct genets, 59 were represented by a single tree or ramet.

#### Genetic Relationship Among Genets

**Figure 4:**
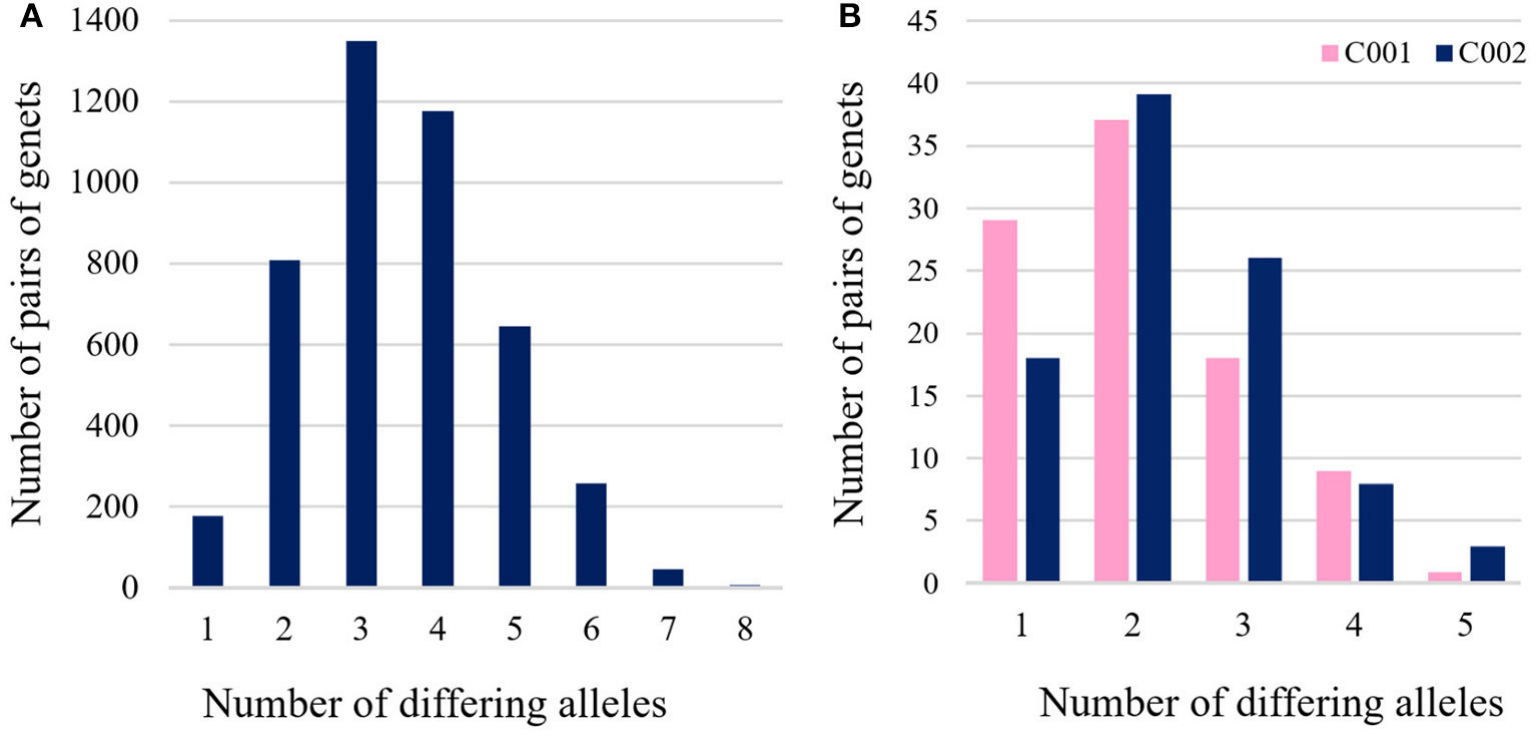
**(A)** Histogram of pairwise distances (number of different alleles) based on the 12 polymorphic microsatellites among 95 ‘Galega vulgar’ genets and **(B)** histogram of pairwise distances based on the 12 polymorphic microsatellites among the two most frequent genets (C001 and C002) and the rest of genets.

The pairwise genetic distance (D_psa_) based on the 12 polymorphic microsatellites was calculated among the 95 genets and ranged from 0.04 (one differing allele out of 24) to 0.33 (eight differing alleles) (Figure 4A). The two most frequent genets (C001 and C002) differed only in a single allele (M05, Table 2). When comparing the two most frequently found genets (C001 and C002) with the remaining 93 genets, distances range from 0.04 (one differing allele) to 0.21 (five differing alleles) (Figure 4B).

**Figure 5:**
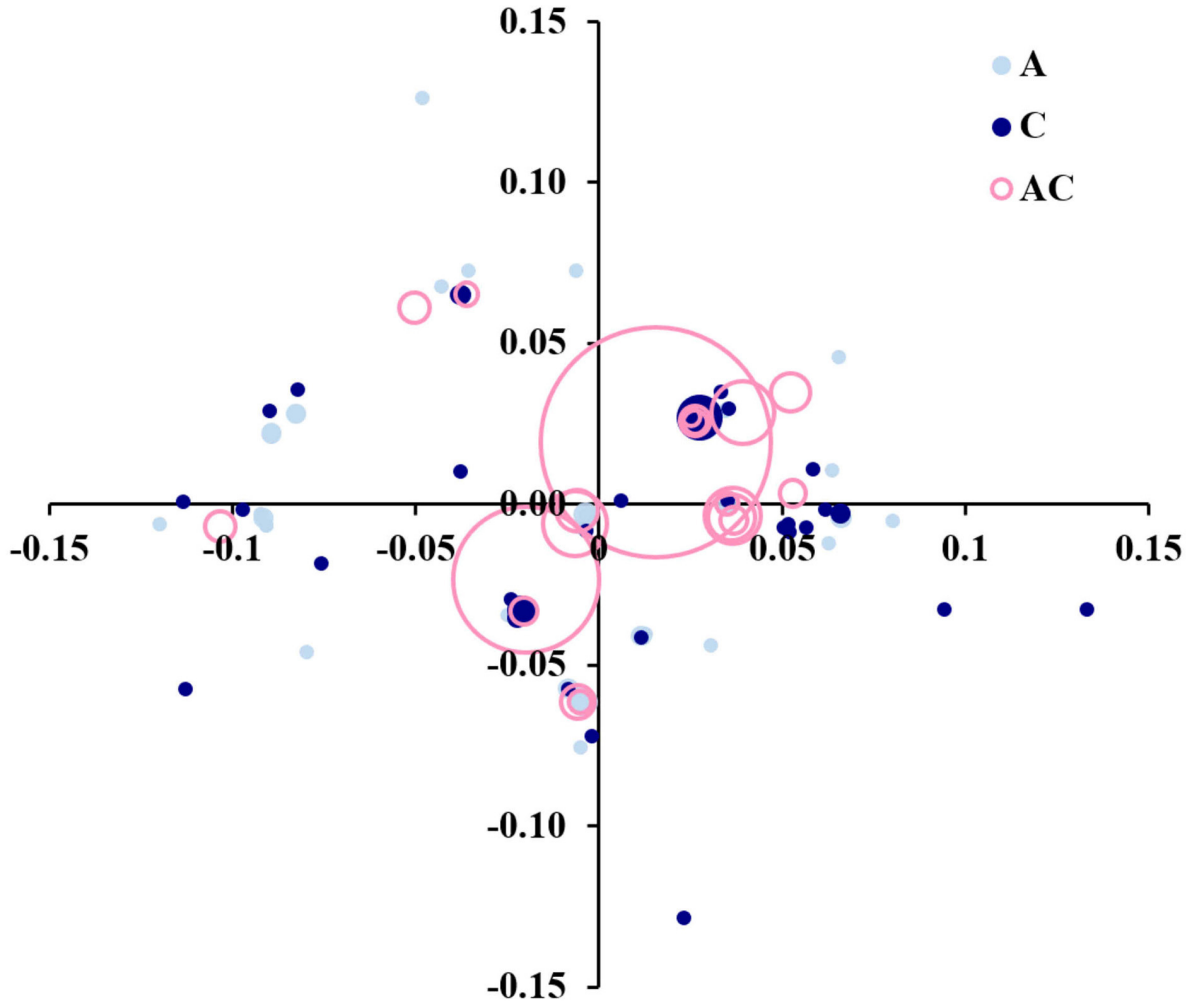
Principal coordinate analysis (PCoA) biplot based on the proportion-of-shared-alleles distance matrix among 95 genets identified by screening 595 ‘Galega vulgar’ trees, with 12 microsatellite markers. The first two coordinates represent 47.10% of the total genetic variability. Circles size proportional to the number of trees sharing the identical genet. Solid light blue circles represent private genets to ancient germplasm, solid dark blue circles private genets to centennial germplasm, and empty pink circles genets shared by both germplasm groups.

A principal coordinate analysis (PCoA) based on the proportion-of-shared-alleles distance was performed to graphically depict the genetic relationships among genets (Figure 5). The first two coordinates represented 47.10% of the total genetic variability. The most frequent genets, represented by the largest circles, are in the center. Most of the trees were clustered in a few genets, with a mixture of ancient and centennial germplasm (pink empty circles in Figure 5). Still, it was possible to identify some other smaller differentiated clusters comprising different genets of ‘Galega vulgar’ intra-variability. Additionally, some unique and diverse genotypes (solid light blue or solid dark blue circles in Figure 5) that represented genets not shared between the two groups of germplasm (ancient and centenary) were also identified.

#### Genet vs. Endocarp Profile

After identifying the 95 distinct genets of ‘Galega vulgar’ and taking into consideration the existence of four endocarp profiles in this variety, the correlation between the two variables was calculated. No significant correlation was obtained (*r* = −0.00) (as shown in Supplementary Figure 1). The same absence of correlation happened when considering only ancient trees (*r* = −0.03) (Supplementary Figure 2). Indeed, each of the four endocarp types could be found in many different genets; in addition, each genet could be associated with more than one type of endocarp. As examples, 66 different genets showed endocarp' profile 1 (“rounded base” with a “0.30–0.45 g weight”), 23 genets profile 2 (“rounded base” and “ <0.30 g weight”), 43 profile 3 (“acute base” and “0.30–0.45 g weight”), and 7 profile 4 (“acute base” and “ <0.30 g weight”), showing the two most frequent genets (C001 and C002), all of the four endocarp profiles.

### ‘Galega Vulgar’ Intra-Varietal Diversity

#### Centenary vs. Ancient Germplasm

Regarding the comparison of overall centennial and ancient trees and between age groups (C1, A1, A2, and A3), no group differed significantly in any of the standard genetic diversity parameters (*N*_*ar*_*, H*_*O*_*, H*_*E*_) (Table 3). However, 12, 7, and 8 private alleles were detected in C1, A1, and A2 groups, respectively. Thus, the overall level of diversity was maintained across time, but the maintained diversity has changed, since the alleles in ancient trees were not the same as in centennial trees. Additionally, as shown in Table 3, the number of multi-locus genotypes (*N*_*c*_) identified in the centenary and ancient germplasm groups was also similar (57 vs. 58), but the clonal richness (*N*_*cr*_) was higher in ancient trees (48.94 vs. 58.00). Among age groups, the clonal richness was higher in A1 and A3 groups (5.58 and 5.00, respectively). The number of private clones (*N*_*pc*_) in the two germplasm groups (centenary vs. ancient) was similar (37 vs. 38) but the private clonal richness was higher in ancient trees (30.07 vs. 38.00), with the highest value in A1 group. Regarding the genotypic richness (or clonal diversity; *R*), it was higher in ancient trees (0.17 vs. 0.22), assuming the highest value in the oldest A3 group. Consequently, the probability of encountering distinct genets when randomly taking two trees of an age group (D^*^) was also slightly higher in ancient trees (0.77 vs. 0.79). In conclusion, some specific alleles or genets of each age group were different (different private alleles and different private clones), with the number of allele combinations higher on ancient genotypes, which translates into the loss of some allele combinations across time.

**Table 3:** Allelic, clonal, and genetic diversity between 335 centennial and 260 ancient ‘Galega vulgar’ trees and among age groups (age groups C1 from 80 to 100 years old, A1 from 400 to 599 years old, A2 from 600 to 900 years old, and A3 more than 1,000 years old), using 12 SSRs.

**Parameter**	**Germplasm group**		***P*-value**	**Age group**				***P*-value**
	**Centenary**	**Ancient**		**C1**	**A1**	**A2**	**A3**	
*n*	335	260	n/a	335	109	143	8	n/a
**Allelic diversity**								
*N_*av*_*	4.75	5.17	n/a	4.75	3.75	4.25	2.00	n/a
*N_*ar*_*	4.44	5.17	0.16	2.32	2.22	2.33	2.00	0.11
*N_*pra*_*	12	17	n/a	12	7	8	0	n/a
**Clonal diversity**								
*N_*c*_*	57	58	n/a	57	36	34	5	n/a
*N_*cr*_*	48.94	58.00	n/a	4.78	5.58	4.35	5.00	n/a
*n_*c*(*C*001)_*	148	106	n/a	148	36	67	3	n/a
*n_*c*(*C*001)_ (%)*	44.18	40.77	n/a	44.18	33.03	46.85	37.50	n/a
*n_*c*(*C*002)_*	54	50	n/a	54	18	30	2	n/a
*n_*c*(*C*002)_ (%)*	16.12	19.23	n/a	16.12	16.51	20.98	25.00	n/a
*N_*pc*_*	37	38	n/a	37	15	19	0	n/a
*n_*c*(*pc*)_*	55	50	n/a	55	19	20	0	n/a
*n_*c*(*npc*)_ (%)*	16.42	19.23	n/a	16.42	17.43	13.99	0.00	n/a
*N_*pcr*_*	30.07	38.00	n/a	1.29	1.38	1.12	0.00	n/a
*R*	0.17	0.22	n/a	0.17	0.32	0.23	0.57	n/a
D^*^	0.77	0.79	n/a	0.77	0.86	0.74	0.86	n/a
*V*	0.69	0.68	n/a	0.69	0.72	0.58	0.56	n/a
**Genetic diversity**								
*H_*O*_*	0.67	0.66	0.08	0.67	0.66	0.66	0.67	0.40
*H_*E*_*	0.43	0.42	0.71	0.43	0.40	0.43	0.37	0.09

n, total sample size; N_av_, average number of alleles per locus; N_ar_, allelic richness; N_pra_, number of private alleles; N_c_, number of multi-locus genotypes (i.e. distinct genotypes); N_cr_,, clonal richness; n_c(C001)_, clonal size of the multi-locus genotype (genet) C001; n_c(C001)(%)_,, percentage of the samples belonging to the genet C001; n_c(C002)_, clonal size of the multi-locus genotype (genet) C002; n_c(C002)(%)_,, percentage of the samples belonging to the genet C002; N_pc_, number of private clones; n_c(pc)_,, total clonal size of the private clones; n_c(npc)(%)_, percentage of the samples belonging to the private clones; N_pcr_, private clonal richness; R, genotypic richness; D^*^, Simpson's complement index; V, Simpson's evenness index; H_O_, observed heterozygosity; H_E_, expected heterozygosity; n/a, not applicable.

Comparing the observed and expected heterozygosity (*H*_*O*_ and *H*_*E*_), no significant differences were found between centenary and ancient germplasm groups (*H*_*O*_ = 0.67 vs. 0.66; *H*_*E*_= 0.43 vs. 0.42, in centennial and ancient olive trees, respectively), neither among age groups. In all the cases, the observed heterozygosity was higher than the expected heterozygosity (Table 3).

Out of the 95 identified genets, only 20 (21.05%) were identified in both germplasm groups. Nevertheless, the six most frequent genets (C001-C006), represented by 426 (71.60%) out of 595 sampled trees, were found in both germplasm groups. By analyzing the genets with unique age individuals (centennial only vs. ancient only) (Table 4), no significant differences were found in the calculated genetic diversity parameters (*N*_*ar*_*, H*_*O*_*, H*_*E*_). Even so, the unique ancient group presented the highest values for allelic richness (4.33 vs. 4.95), clonal richness (34.25 vs. 38.00), private clonal richness (34.25 vs. 38.00), R (0.68 vs. 0.76), D^*^ (0.96 vs. 0.99) and V (0.72 vs. 0.88). Regarding the observed and expected heterozygosity, both were higher in the centennial group.

**Table 4:** Allelic, clonal, and genetic diversity among ‘Galega vulgar’ clones with unique age individuals, using 12 SSRs in 595 individual trees.

**Parameter**	**Germplasm group**		***P*-value**
	**Centenary**	**Ancient**	
*n*	55	50	n/a
**Allelic diversity**			
*N_*av*_*	4.33	5.00	n/a
*N_*ar*_*	4.33	4.95	0.30
*N_*pra*_*	12	20	n/a
**Clonal diversity**			
*N_*c*_*	37	38	n/a
*N_*cr*_*	34.25	38.00	n/a
*N_*pc*_*	37	38	n/a
*n_*c*(*pc*)_*	37	38	n/a
*n_*c*(*npc*)_ (%)*	100	100	n/a
*N_*pcr*_*	34.25	38.00	n/a
*R*	0.67	0.76	n/a
D^*^	0.96	0.99	n/a
*V*	0.72	0.88	n/a
**Genetic diversity**			
*H_*O*_*	0.68	0.67	0.08
*H_*E*_*	0.45	0.44	0.68

n, total sample size; N_av_, average number of alleles per locus; N_ar_, allelic richness; N_pra_, number of private alleles; N_c_, number of multi-locus genotypes (i.e. distinct genotypes); N_cr_,, clonal richness; N_pc_, number of private clones; n_c(pc)_, total clonal size of the private clones; n_c(npc)(%)_, percentage of the samples belonging to the private clones; N_pcr_, private clonal richness; R, genotypic richness; D^*^, Simpson's complement index; V, Simpson's evenness index; H_O_, observed heterozygosity; H_E_, expected heterozygosity; n/a, not applicable.

In this study, we did not find any evidence for cases of homonymy (no genotype was genetically completely different), nor for sexual propagation (no genotype had a high number of differing alleles from the other genotypes) that could be explained as a result of out-crossing events, or by a selection pressure made by olive growers to maintain a ‘Galega vulgar’ morphotype.

#### Endocarp Profile vs. Genetic Diversity

By analyzing the intra-varietal diversity of all validated ‘Galega vulgar’ samples, significant differences were found in allelic richness (*P* < 0.05) among the four endocarp profiles. A Tukey's HSD test (between all the four profiles E1, E2, E3, and E4) revealed that the E1 and E4 profiles were significantly different from each other but not from E2 and E3 profiles. For clonal richness (*N*_*cr*_), private clonal richness (N_*pcr*_) and expected heterozygosity (*H*_*E*_), comparing all the endocarp profiles, E2 presented the highest values (11.93, 3.25, and 0.43, respectively), and E4 the lowest values (7.00, 1.00, and 0.39, respectively) (Table 5).

**Table 5:** Allelic, clonal, and genetic diversity among the four different ‘Galega vulgar’ endocarp profiles, using 12 SSRs in 595 individual trees.

**Parameter**	**Endocarp profile**				***P*-value**
	**E1**	**E2**	**E3**	**E4**	
*n*	350	59	162	24	n/a
**Allelic diversity**					
*N_*av*_*	5.25	3.33	4.50	2.17	n/a
*N_*ar*_*	2.52^a^^*^	2.47^ab^^*^	2.48^ab^^*^	2.17^b^^*^	0.03
*N_*pra*_*	16	1	9	0	n/a
**Clonal diversity**					
*N_*c*_*	66	24	43	7	n/a
*N_*cr*_*	10.12	11.93	10.96	7.00	n/a
*n_*c*(*C*001)_*	149	25	68	12	n/a
*n_*c*(*C*001)_ (%)*	42.57	42.37	41.98	50.00	n/a
*n_*c*(*C*002)_*	66	6	28	4	n/a
*n_*c*(*C*002)_ (%)*	18.86	10.17	17.28	16.67	n/a
*N_*pc*_*	38	8	18	1	n/a
*n_*c*(*pc*)_*	45	8	20	3	n/a
*n_*c*(*npc*)_ (%)*	12.86	13.56	12.35	12.50	n/a
*N_*pcr*_*	3.03	3.25	2.92	1.00	n/a
*R*	0.19	0.40	0.26	0.26	n/a
D*	0.78	0.81	0.79	0.73	n/a
*V*	0.68	0.52	0.64	0.62	n/a
**Genetic diversity**					
*H_*O*_*	0.67	0.67	0.67	0.67	1.00
*H_*E*_*	0.42	0.43	0.42	0.39	0.07

n, total sample size; N_av_, average number of alleles per locus; N_ar_, allelic richness; N_pra_, number of private alleles; N_c_, number of multi-locus genotypes (i.e. distinct genotypes); N_cr_, clonal richness; n_c(C001)_, clonal size of the genet C001; n_c(C001)(%)_, percentage of the samples belonging to the genet C001; n_c(C002)_, clonal size of the genet C002; n_c(C002)(%)_, percentage of the samples belonging to the genet C002; N_pc_, number of private clones; n_c(pc)_, total clonal size of the private clones; n_c(npc)(%)_, percentage of the samples belonging to the private clones; N_pcr_, private clonal richness; R, genotypic richness; D^*^, Simpson's complement index; V, Simpson's evenness index; H_O_, observed heterozygosity; H_E_, expected heterozygosity; n/a, not applicable.

* *Small superscript letters reflect the result from Tukey's HSD test. Different letters in the same row indicate significant differences between values at P < 0.05*.

#### Geographical Location vs. Genetic Diversity

From the comparison of the ten prospected geographical districts (Figure 1), no significant differences were found for *N*_*ar*_, *H*_*O*_, *H*_*E*_ among districts (*P*-value = 0.78, 0.79, 0.73, respectively).

However, looking at the parameters of clonal diversity among these regions (N_*cr*_, N_*pcr*_, D^*^, and V), Portalegre, Santarém, Lisboa, and Évora showed the highest values (exception for Lisboa N_*pcr*_) (Table 6). More specifically, Lisboa had the highest number of different genets (highest clonal richness, N_*cr*_) and therefore the highest probability of encountering two different genets (highest value of D^*^), while Portalegre had the highest number of private genets (highest private clonal richness, N_*pcr*_). Finally, the region having the most equitable clonal distribution, i.e., all the genets had almost the same frequency of tested trees (highest value of V), was Santarém (Table 6).

**Table 6:** Allelic, clonal, and genetic diversity among 595 ‘Galega vulgar’ trees grouped into ten regions and into orchard and isolated trees, using 12 SSRs.

**Location**	***n_***a***_***	***n_***c***_***	**Allelic diversity**			**Clonal diversity**													**Genetic diversity**	
			***N_**av**_***	***N_**ar**_***	***N_**pra**_***	***N_**c**_***	***N_**cr**_***	***n_**c(C001)**_***	***n_**c(C001)**_ (%)***	***n_**c(C002)**_***	***n_**c(C002)**_ (%)***	***N_**pc**_***	***n_**c(pc)**_***	***n_**c(npc)**_ (%)***	***N_**pcr**_***	***R***	D*	***V***	***H_**O**_***	***H_**E**_***
Viseu	6	76	3.00	2.04	1	17	4.68	48	58.54	0	0.00	6	8	9.76	0.94	0.20	0.65	0.49	0.67	0.41
Coimbra	0	10	2.08	2.08	0	5	5.00	6	60.00	1	10.00	1	1	10.00	1.00	0.44	0.67	0.00	0.67	0.39
Castelo Branco	9	37	2.67	2.10	1	14	5.24	18	39.13	11	23.91	5	5	10.87	1.09	0.29	0.79	0.65	0.67	0.42
Leiria	27	22	2.50	1.96	2	10	3.83	31	63.27	8	16.33	4	5	10.20	0.98	0.19	0.58	0.42	0.67	0.40
Portalegre	20	36	3.00	2.18	2	19	5.63	13	23.21	21	37.50	9	12	21.43	1.98	0.33	0.81	0.63	0.67	0.42
Santarém	88	81	4.17	2.09	10	37	5.71	63	37.28	33	19.53	19	29	17.16	1.62	0.21	0.81	0.72	0.66	0.42
Lisboa	28	0	2.67	2.16	0	12	6.01	9	32.14	4	14.29	2	2	7.14	0.71	0.41	0.85	0.67	0.67	0.39
Setúbal	22	10	2.50	2.10	0	13	5.42	17	53.13	1	3.13	4	5	15.63	1.47	0.39	0.72	0.31	0.66	0.41
Évora	60	38	3.83	2.18	8	29	5.84	34	34.69	24	24.49	15	17	17.35	1.72	0.29	0.82	0.68	0.67	0.42
Beja	0	25	2.25	1.98	2	8	4.29	15	60.00	1	4.00	3	3	12.00	1.20	0.29	0.63	0.33	0.68	0.38
*P*-value	n/a	n/a	n/a	0.78	n/a	n/a	n/a	n/a	n/a	n/a	n/a	n/a	n/a	n/a	n/a	n/a	n/a	n/a	0.79	0.73
Orchard	185	330	5.50	3.79	29	84	24.45	222	43.11	95	18.45	69	118	22.91	15.74	0.16	0.78	0.69	0.67	0.43
Isolated	75	5	3.75	3.75	8	26	26.00	32	40.00	9	11.25	11	14	17.50	11.00	0.32	0.82	0.65	0.67	0.42
*P*-value	n/a	n/a	n/a	0.91	n/a	n/a	n/a	n/a	n/a	n/a	n/a	n/a	n/a	n/a	n/a	n/a	n/a	n/a	0.85	0.56

n_a_, number of ancient trees; n_c_, number of centennial trees; N_av_, average number of alleles per locus; N_ar_, allelic richness; N_pra_, number of private alleles; N_c_, number of multi-locus genotypes (i.e. distinct genotypes); N_cr_, clonal richness; n_c(C001)_, clonal size of the genet C001; n_c(C001)(%)_, percentage of the samples belonging to the genet C001; n_c(C002)_, clonal size of the genet C002; n_c(C002)(%)_, percentage of the samples belonging to the genet C002; N_pc_, number of private clones; n_c(pc)_, total clonal size of the private clones; n_c(npc)(%)_, percentage of the samples belonging to the private clones; N_pcr_, private clonal richness; R, genotypic richness; D^*^, Simpson's complement index; V, Simpson's evenness index; H_O_, observed heterozygosity; H_E_, expected heterozygosity; n/a, not applicable.

Regarding the comparison between orchard and isolated trees, no significant differences were found for *N*_*ar*_, *H*_*O*_, *H*_*E*_ (*P*-value = 0.91, 0.85, 0.56, respectively) (Table 6). Nevertheless, orchard trees had the highest values for private clonal richness (15.74 vs. 11.00), and Simpson's evenness index, V (0.69 vs. 0.65), while isolated trees revealed the highest clonal richness (26 vs. 24.45) and consequently, the highest Simpson's complement index, D^*^ value (0.82 vs. 0.78).

Analysis of molecular variance (AMOVA) was performed to test the existence of genetic structure among and within groups: germplasm groups (centenary vs. ancient), age groups (C1, A1, A2, and A3), endocarp profile groups (E1, E2, E3, and E4), prospected geographical districts groups, and tree location groups (orchard vs. isolated tree). Negative values of ϕ-statistics were observed in both tests, suggesting the absence of any genetic structure (Table 7).

**Table 7:** Analysis of molecular variance for the partitioning of genetic diversity among and within groups (germplasm, age, endocarp profiles, geographical districts, and tree location groups), using 12 SSRs in 595 ‘Galega vulgar’ trees.

**Analysis**	**Source of variation**	**df**	**Variance components**	**% Total variation**	**ϕ-statistics**	***P(ϕ)***
(A)	Among germplasm groups	1	−0.02	−0.68	−0.01	0.91
	Within germplasm groups	228	2.81	100.68		
(B)	Among age groups	3	−0.03	−1.25	−0.01	0.97
	Within age groups	260	2.53	101.25		
(C)	Among endocarp profiles	3	−0.03	−1.35	−0.01	1.00
	Within endocarp profiles	276	2.54	101.35		
(D)	Among districts	9	−0.06	−2.27	−0.02	0.79
	Within districts	318	2.77	102.27		
(E)	Among tree location	1	−0.02	−0.96	−0.01	0.93
	Within tree location	218	2.56	100.96		

*(A), among and within germplasm groups (centenary vs. ancient); (B), among and within age groups (C1/A1/A2/A3); (C), among and within endocarp profiles (E1/E2/E3/E4); (D), among and within the 10 prospected geographical districts; (E), among and within the tree location (orchard vs. isolated tree)*.

#### Putative Ancestral Origin of Genets Diversity

Values for the correlation coefficients calculated to analyze the ancestral origin of this variety after identifying the presence of one main genet (C001), by clarifying the relationship between the amount of clonal divergence and clonal size (number of ramets), can be found in Supplementary Figures 3–5.

The net divergence, calculated by summing up the number of different alleles of a genet to all the other genets in the analysis, ranged from 198 to 523, with an average of 330. The most frequent genet (C001) had the lowest net divergence (198), followed by the second most frequent genet (C002) with 221. A negative correlation (*r* = −0.49; *P* < 0.0001) was observed between the net divergence of the genets and their number of ramets (Supplementary Figure 3). A very strong positive correlation (*r* = 0.91; *P* < 0.0001) was observed between the number of differentiating alleles of genets in comparison to the most frequent genet (C001) and their net divergence (Supplementary Figure 4). Similarly, a negative correlation (*r* = −0.39; *P* < 0.001) was observed between the number of differentiating alleles of genets in comparison to the most frequent genet (C001) and their number of ramets. The other most frequent genets (C002–C008) had a single allele difference from C001 (Supplementary Figure 5).

**Figure 6:**
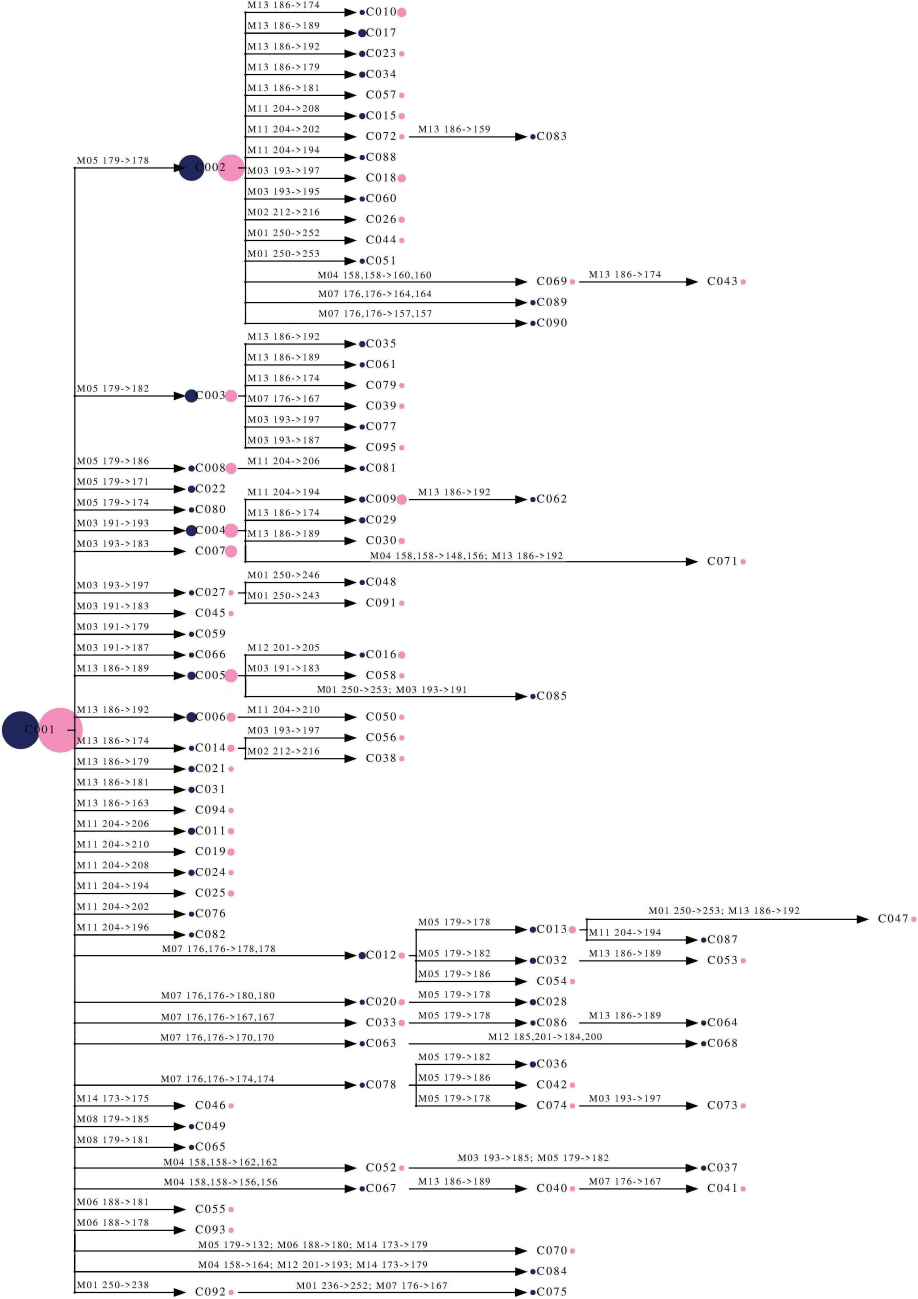
Minimum spanning tree of 95 ‘Galega vulgar’ genets based on the matrix of genetic distances computed as the number of different alleles among genets. The tree was rooted using the most frequent genet (C001) as outgroup. The circles are proportional to the number of trees belonging to each genet (number of ramets). Blue circles represent ancient trees, while the pink represent centennial trees. The numbers (M01–M14) on the branches refer to microsatellite loci (as stated in Table 2) and the allelic substitution that gave rise to a novel multi-locus genotype (genet).

By taking into consideration all of these correlations and using the information on all the allelic substitutions that gave rise to a novel genet, we proposed a minimum spanning tree for ‘Galega vulgar’, based on the matrix of genetic distances and computed as the number of different alleles among genets (Figure 6). With this tree, we intended to depict a complete genealogy/pedigree of all ‘Galega vulgar’ genets analyzed.

## Discussion

To clarify the genetic diversity evolution, potential ancestral origin, and overall genetic relationships within the national heritage germplasm of ‘Galega vulgar’, 595 olive trees belonging to different age groups were characterized, using 12 SSR markers, validated as ‘Galega vulgar’ variety by endocarp measurements. To the best of our knowledge, this survey represents the first attempt of a molecular characterization in the ‘Galega vulgar’ variety or any other *Olea* variety with such a wide representative sample of individuals, combining both ancient and centennial trees, in the Portuguese territory.

Ninety-five distinguishable genets were identified within the collection of analyzed trees, differing in up to eight alleles, and revealing the presence of a reasonable amount of intra-variability among the ‘Galega vulgar’ variety. However, by examining the principal coordinate analysis (PCoA) results based on the proportion-of-shared-alleles distance matrix, we also observed that the majority of the individual trees were clustered in a few genets, with a mixture of undistinguishable ancient and centennial germplasm. Similar findings were observed for other Portuguese varieties, namely ‘Verdeal-Transmontana’ and ‘Cobrançosa’, that display a wide intra-varietal genetic variability (Gomes et al., 2008; Martins-Lopes et al., 2009). The 95 genets were also characterized by some morphological diversity depicted by the presence of four different ‘Galega vulgar’ endocarp profiles, with the profile characterized by a rounded base shape in position A and a weight of 0.30–0.45 g, the most common among all the genets. No correlation was found between the different ‘Galega vulgar’ genets and the four endocarp profiles, not even when comparing only the ancestral trees. Usually, an olive variety is characterized by a unique endocarp profile. However, for the ‘Galega vulgar’ variety, well-accepted published criteria define four different profiles, all found among the present analyzed trees. As hypothesized by Trujillo et al. (2014), morphological changes could occur without affecting the amplified SSR region due to punctual somatic mutations, especially if we are in the presence of co-ancestral origin of varieties. Thereby, we hypothesized that somatic mutations might have occurred and led to morphological variation in the endocarps, without affecting the analyzed SSR profiles. On the other hand, we identified different genets with the same endocarp profile. One hypothesis that could potentially explain this observation is that the genomic regions with SSRs can accumulate somatic mutations without affecting phenotypic traits, since these regions are neutrally evolving and highly variable (Díez et al., 2011). Nevertheless, a revision of the identification criteria for the variety ‘Galega vulgar’ may be necessary, since the current criteria leads to a diversity of endocarp types not common in other olive varieties.

The majority of the ‘Galega vulgar’ genotypes clustered in two (C001 and C002) very frequent and genetically similar genets. The same results were already described for other Portuguese varieties, as ‘Verdeal-Transmontana’, where the analyzed trees fell into two major genotypic groups (Gomes et al., 2008). In our study, the two most frequent genets, differing in just one allele, were present in all the four endocarp profiles of ‘Galega vulgar’ and represented 60.17% of all the trees. Moreover, taking into consideration the genets with a one-allele difference (29 genets) and a two-allele difference (37 genets) from the most frequent genet, the total would be around 94% of all the analyzed ‘Galega vulgar’ trees. Although there are up to eight differing alleles among some genets, the maximum distance from the two most frequent genets is lower (i.e., five differing alleles out of 24). This suggests that, together with the very strong positive correlation between the number of differentiating alleles of genets in comparison with the most frequent genet (C001) and their net divergence, we are in the presence of an ancestral monoclonal origin of the ‘Galega vulgar’ variety, with genet C001 as the most likely origin of all the investigated genets. Moreover, a weak negative correlation was observed between the number of differentiating alleles of genets in comparison with the most frequent genet and their clonal size, or their number of ramets. With these results, we depicted a complete genealogy/pedigree of all ‘Galega vulgar’ genets of this study, representing the ‘Galega vulgar’ intra-varietal diversity found in a minimum spanning tree. Our suggestion of an ancestral monoclonal origin of ‘Galega vulgar’ contradicts what was proposed by Gemas et al. (2004), analyzing a much smaller sample of ‘Galega vulgar’ trees. In this other study, the genetic diversity of ‘Galega vulgar’ is explained by its polyclonal origin. Even recognizing that the detected small differences between genotypes could be due to somatic mutations, Gemas et al. (2004) stated that mutations cannot account for all the diversity found. Only the combination of the occurrence of somatic mutations and the possibility of the incorporation of individuals originated by sexual propagation into the stock used for vegetative propagation explained the polyclonal origin of ‘Galega vulgar’. In our study, since 94% of analyzed trees showed only a two-allele difference, it is more likely that the genotypic and phenotypic diversification of ‘Galega vulgar’ occurred due to somatic mutations across time rather than a polyclonal origin. Supporting this hypothesis, several genetic diversity studies already pointed to a monoclonal origin for several olive tree varieties, with reports of up to five different alleles (Khadari et al., 2008; Muzzalupo et al., 2010; Strikić et al., 2011). Nevertheless, it is also possible that a number of the identified genets are first- or second-degree relatives. Indeed, since the two main genets (C001 and C002) differ only in one allele, the two ortets, i.e., the original plants from which the members of a clone are descended, could be considered as phenotypically very similar full siblings derived from the cross between two, in turn, closely related parental trees. Still, further studies are needed to disentangle the various factors affecting the evolution of propagated species and to understand how far somatic mutations can change the varieties (Díez et al., 2011). In addition, the extent, nature, and implications of somatic mutations due to vegetative propagation is also poorly understood (McKey et al., 2010). By cytoplasmatic markers analysis it was hypothesized that ‘Galega vulgar’ belongs to a lineage originated in Eastern Mediterranena area (including Cyprus) (Besnard et al., 2013b). In the future, it would be fruitful to verify if all the 595 individuals from this study have the same cytoplasm and compare the results with other reference studies, to better clarify the ‘Galega vulgar’ origin.

In our work, the observed heterozygosity (*H*_*O*_) was higher than the expected heterozygosity (*H*_*E*_) in both germplasm groups (centenary and ancient), and also considering the different age groups (C1, A1, A2, and A3). Using SSR markers, several authors also reported the same trend in different olive germplasm (Díaz et al., 2006; Poljuha et al., 2008; Erre et al., 2010) and this could be explained by the maintenance of early generation admixed individuals (Besnard, 2016) or even the accumulation of somatic mutations on highly mutable loci in ancient genotypes, as reported by Baali-Cherif and Besnard (2005) and Barazani et al. (2014).

Differences were found among prospected geographical districts regarding clonal diversity, with the regions of Portalegre, Santarém, Lisboa, and Évora the ones presenting higher diversity parameter values. According to Gemas et al. (2004), the “Ribatejo-Santarém” region was indicated as the ecological region of origin and dispersion of ‘Galega vulgar’, due to the higher genetic diversity found. Comparing the agroecological regions considered by Gemas et al. (2004) with the ten prospected geographical districts of our study, the Santarém district is a mixture of the “Ribatejo-Abrantes” and “Ribatejo-Santarém” agroecological regions and the Lisboa district is included in the “Ribatejo-Santarém” agroecological region. And so, partially, our findings are in accordance with the ones reported by Gemas et al. (2004) on the probable origin of dissemination of ‘Galega vulgar’. However, in our study, the Portalegre and Évora districts also presented high clonal diversity values. In Gemas et al. (2004) these districts are included in the “Alto Alentejo” agroecological region, which was reported as having the lowest values of genetic diversity. A possible explanation for these differences resides in the fact that the *ex-situ* ‘Galega vulgar’ collection used by Gemas et al. (2004) [established by Martins et al. (1997)] had only 13 individuals of the “Alto Alentejo” region, and this might be an under-representation of the diversity still existing in that region.

The comparative analysis between centennial and ancient germplasm revealed that there was an effective loss of some allelic combinations from ancestral to centennial trees, although this was not associated with a decrease of overall allelic diversity. On one hand, the allelic richness, as well as the gene diversity, were similar in the four age groups (probably due to an accumulation of somaclonal variation that controverted any eventual loss of alleles), being the most frequent genets present in centennial orchards and therefore still conserved under production. On the other hand, the ancient trees showed a higher clonal richness and private clonal richness (especially A1 group) compared with the centennial trees, meaning that some allele combinations were lost across time, representing a case of genetic erosion. Indeed, Maxted and Guarino (2006) described the loss of some combination of alleles over time as a possible phenomenon of genetic erosion. Also, Besnard et al. (2013a) revealed genetic erosion in the process of domestication by studying the patterns of genetic differentiation in the Mediterranean cultivars and wild olive trees from the Mediterranean basin and the Saharan mountains with nuclear microsatellite and plastid DNA. The negative values of ϕ-statistics in our work suggests the absence of genetic structure considering all the groups. This led us to infer that the loss of some less frequent genets present in ancient trees was completely random and, over a certain time frame, more similar to a normal stochastic process of drift (of genets but not alleles) and not a sort of genetic bottleneck. In this way, a pair of genets belonging to the same germplasm or age group is not genetically more similar than a pair of genets belonging to the other germplasm or age group.

However, it is important to mention that estimating the age of live olive trees represented a very challenging task. According to Lavee (1996), the inner and oldest part of olive tree wood decays in older trees, being impossible to identify the center of origin and, therefore, the exact dating of ancient trees. Moreover, many independent trunks can replace the original single tree trunk, and different factors may affect plant growth and wood decay, which may result in different growth speeds and distort interpretations of tree age by its annual rings (Ninot et al., 2018). Nevertheless, several studies pointed out the importance of algorithms based on trunk size to estimate age of olive trees (Michelakis, 2002; Díez et al., 2011; Arnan et al., 2012). In this sense, although the methodology for age classification used in this study was not the most accurate (the age of the sampled trees was roughly calculated based on the trunk diameter and an extrapolation based on a theoretical annual growth rate), it allowed to group and order our samples based on their average age, although with a possible over-estimation of the tree age.

The identified genetic erosion highlights the need to recover the lost diversity at the more recently established orchards. In fact, isolated ancient trees showed a higher clonal richness compared to orchard trees. Therefore, the inclusion of ancient private genets in breeding programs, broadening the programs' genetic basis is a key aspect, since these ancient genets might have interesting traits for survival adaptation due to their longevity (Baldoni et al., 2006; Ninot et al., 2018). Consequently, investing in the protection of these resources *in situ* in particular regions presenting higher diversity could be a way to recover from this genetic erosion.

In a breeding program context, the ‘Galega vulgar’ variety seems to hold considerable potential for selection in view of its morphological diversity (measured here by the diversity in size and shape of endocarps). Indeed, according to Belaj et al. (2012), the WOGB representative sample of ‘Galega vulgar’ (belonging to the most frequent genet identified in this study) is a mixture between the West and Central Mediterranean genetic clusters, deduced from its intermediate position on a multivariate analysis based on molecular and agronomic traits, and where interesting novel combinations of traits might be found.

The morphological diversity with the simultaneous consideration of rounded to acute endocarp shapes and medium to small endocarp weights in the ‘Galega vulgar’ variety could be a sign of an ongoing domestication process or the effect of a long-term clonal propagation process. According to Fuller (2018), in the domestication process of olive trees, fruits and seeds tend to be larger with more pointed (acute to acuminate) ends in cultivated forms, in comparison with wild forms. In our findings, the endocarp profile with higher related wild traits (rounded shape and smaller weight, endocarp profile 2) revealed higher clonal richness, private clonal richness, and genetic diversity, supporting a potential loss of genetic diversity over time. Nevertheless, it is also known that olive varieties are typically clonal propagated, and this technique decreases the number of generations between the varieties and their wild ancestors, and, consequently, the differences between them (McKey et al., 2010; Miller and Gross, 2011). Additionally, clonal propagation facilitates the existence of overlapping generations, which also contributes to this small differentiation (Díez et al., 2016). Corroborating this hypothesis, the ‘Galega vulgar’ from WOGB in the work of Díez et al. (2015), analyzing a data set of wild and cultivated olive trees with SSRs to estimate its most probable demographic model, belongs to a Central cluster that shows signs of a mixture between cultivated and wild olives. So, it is possible that ‘Galega vulgar’ still holds undomesticated forms, which might be beneficial for the selection of interesting phenotypes [such as resistant traits studied by Jiménez-Fernández et al. (2016) and Palomares-Rius et al. (2019) in wild olive trees] in breeding programs. As shown in Lazović et al. (2018), several east Adriatic olive tree varieties revealed many differences in their intra-varietal diversity. It seems that the intra-variability of a certain variety depends on many different factors including a range of contingent historical events. Therefore, the intra-variability findings of this study are specific to the ‘Galega vulgar’ variety. What became clear from our study was that what is important is to have reliable measures to quantify intra-varietal diversity, checked in a sample of adequate size, to allow a proper comparison of the results with other diverse olive tree varieties.

## Conclusions

This study allowed us to track, for an eventual recovery, the genetic diversity still existing in Portugal but that might already be lost in the present ‘Galega vulgar’ orchards and unraveled the ancestral origin of this variety. The inclusion of the ancient private genets (which might have interesting traits for survival adaptation) in breeding programs will broaden their present genetic basis. Moreover, investment should be made in the protection of these resources *in situ* in regions with the highest diversity, and eventually *ex situ*, which might allow genetic studies under the same environmental conditions. As far as we know, this was the most representative study made so far in Portugal regarding the ‘Galega vulgar’ diversity and is a conscious first effort to efficiently preserve and use this national high-quality genetic resource. Moreover, this approach has the potential to be replicated in other diverse varieties, either due to their intermediate nature between different gene pools or due to the presence of a mixture of cultivated and wild traits, as is the case of ‘Galega vulgar’. Thus, it will be possible to accurately access and counteract the risk of further genetic erosion of these varieties, toward their efficient use in breeding programs.

## Data Availability Statement

The datasets presented in this study can be found in online repositories. The names of the repository/repositories and accession number(s) can be found at: https://figshare.com/, doi: 10.6084/m9.figshare.12666446.

## Author Contributions

HS performed the prospection of the individual trees, morphological endocarp characterization, DNA isolation, the SSR genotyping, participated in the analysis of the molecular data, and drafted the manuscript. ZS performed the statistical analysis of the molecular data, and critically participated in the manuscript revision. MLA participate in the analysis of the molecular data and revised the manuscript critically. PF was involved in the prospection phase of this study and revised the manuscript critically. JN revised the manuscript critically. MCVP designed and coordinated the study and participated in the drafting and revising of the manuscript. All authors read and approved the final manuscript.

## Conflict of Interest

The authors declare that the research was conducted in the absence of any commercial or financial relationships that could be construed as a potential conflict of interest.

## Footnotes

^1^Available online at: https://www.internationaloliveoil.org/what-we-do/economic-affairs-promotion-unit/#figures.

^2^Available online at: https://www.gpp.pt/index.php/estatisticas-economicas-e-comercio-internacional/indicadores-do-complexo-agroalimentar-e-florestal.

^3^Available online at: https://cpvo.europa.eu/en/applications-and-examinations/technical-examinations/technical-protocols/cpvo-technical-protocols?t=olive&field_crop_sector_tid=All.

## Supplementary Material

Supplementary Figure 1 Endocarp profile membership of the 95 distinct genets of ‘Galega vulgar’ and correlation analysis between both variables.

Supplementary Figure 2 Endocarp profile membership of the ancient genets of ‘Galega vulgar’ and correlation analysis between both variables.

Supplementary Figure 3 Correlation between the net divergence of 95 ‘Galega vulgar’ genets and their genet size (number of ramets) (logarithmic scale).

Supplementary Figure 4 Correlation between the number of different alleles of 94 ‘Galega vulgar’ genets in comparison to the most frequent genet (C001) and their net divergence.

Supplementary Figure 5 Correlation between the number of different alleles of 94 ‘Galega vulgar’ genets in comparison to the most frequent genet (C001) and their genet size (number of ramets) (logarithmic scale).

Supplementary Table 1 Passport information of the 595 ‘Galega vulgar’ genotypes prospected and evaluated in this study, their endocarp profile and calculated age in years (according to Michelakis, 2002; Koniditsiotis, 2020).

Supplementary Table 2 Agroecological information of the ten geographical districts under study (data collected in the first year of the prospection).
