# Supplementary figures and images for: Accessing Ancestral Origin and Diversity Evolution by Net Divergence of an Ongoing Domestication Mediterranean Olive Tree Variety
Source: Front Plant Sci. 2021 Jun 24;12:688214. doi: 10.3389/fpls.2021.688214 (PMC8265600; doi:10.3389/fpls.2021.688214)

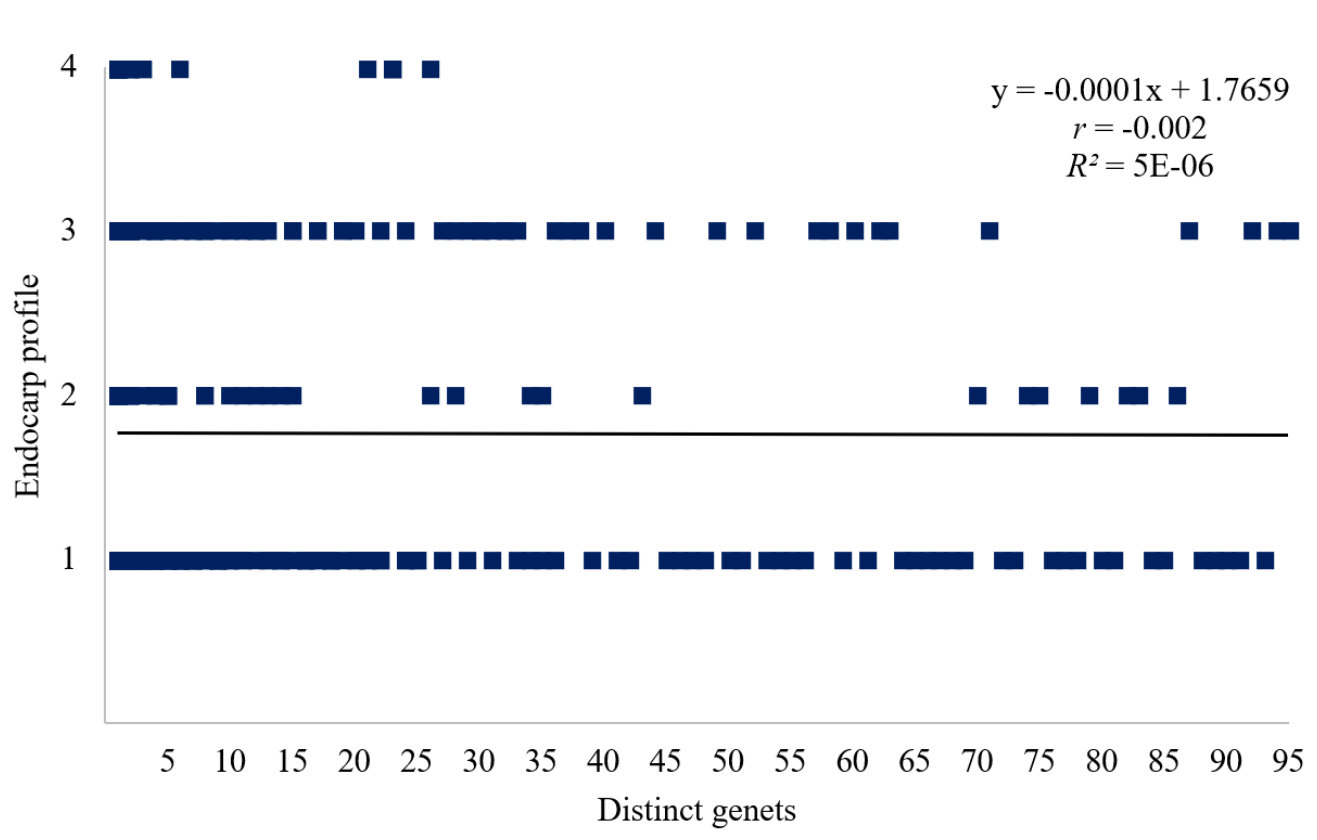

Supplement: Supplementary Figure 1 — Endocarp profile membership of the 95 distinct genets of ‘Galega vulgar’ and correlation analysis between both variables. [file Image_1.JPEG]

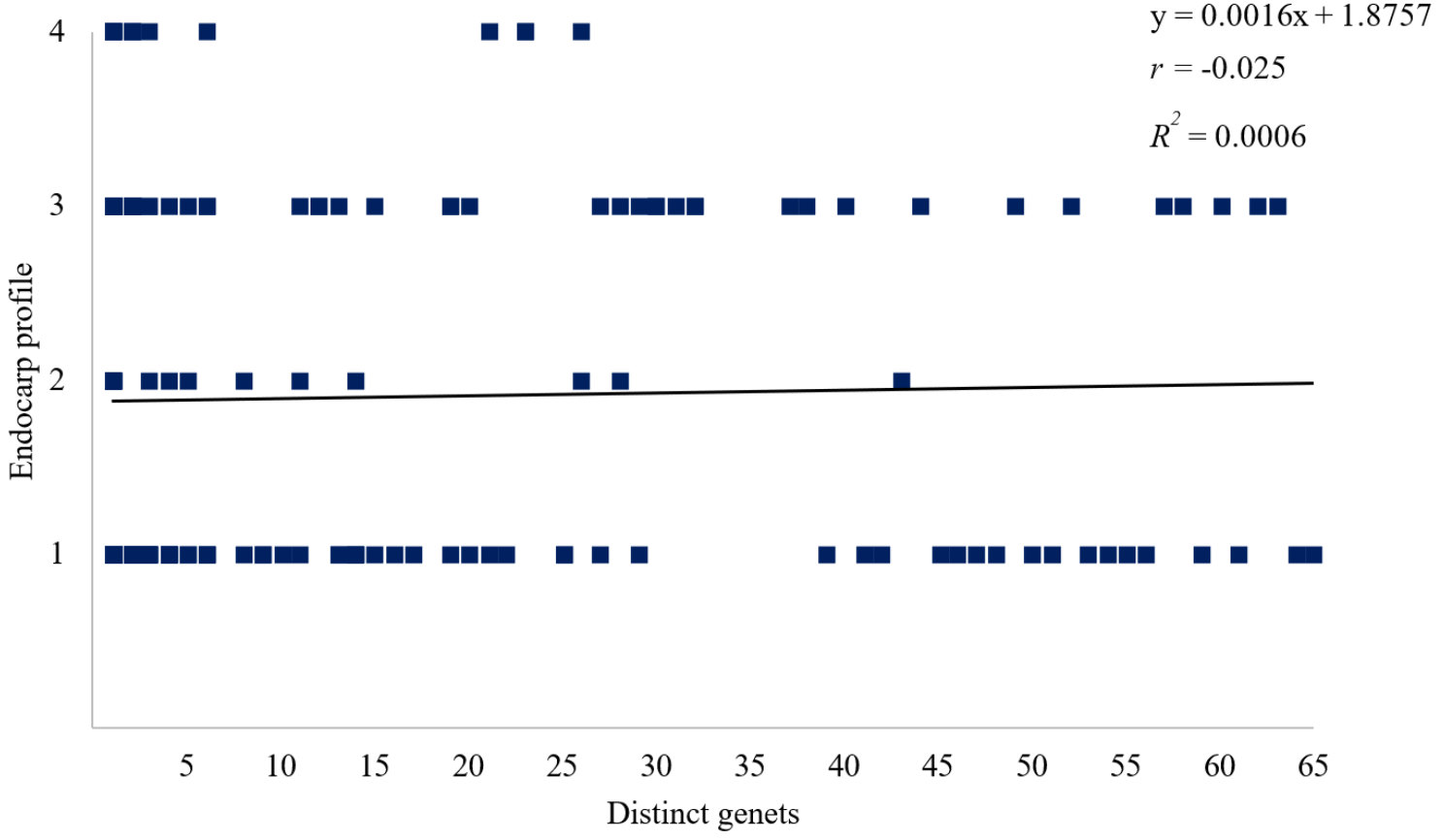

Supplement: Supplementary Figure 2 — Endocarp profile membership of the ancient genets of ‘Galega vulgar’ and correlation analysis between both variables. [file Image_2.JPEG]

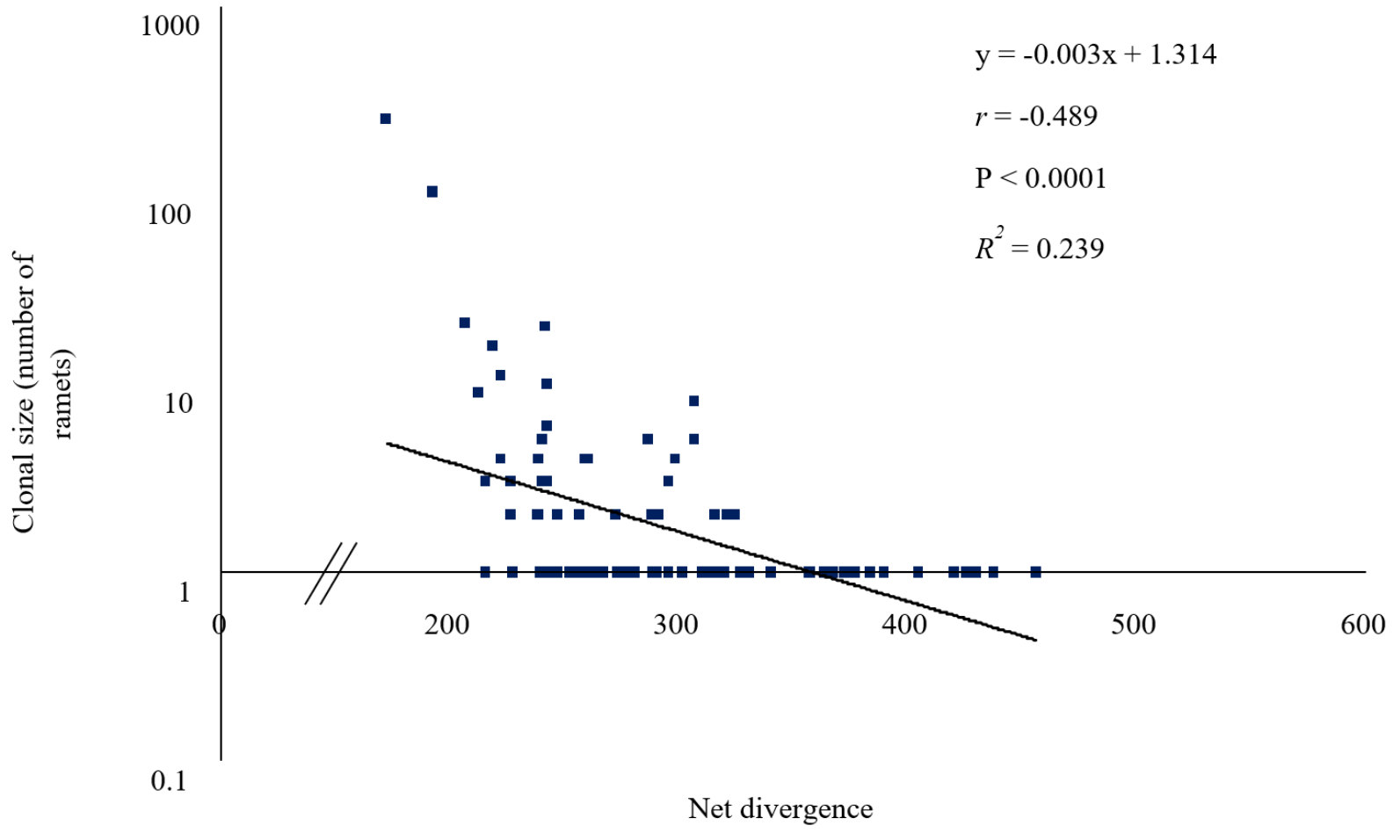

Supplement: Supplementary Figure 3 — Correlation between the net divergence of 95 ‘Galega vulgar’ genets and their genet size (number of ramets) (logarithmic scale). [file Image_3.JPEG]

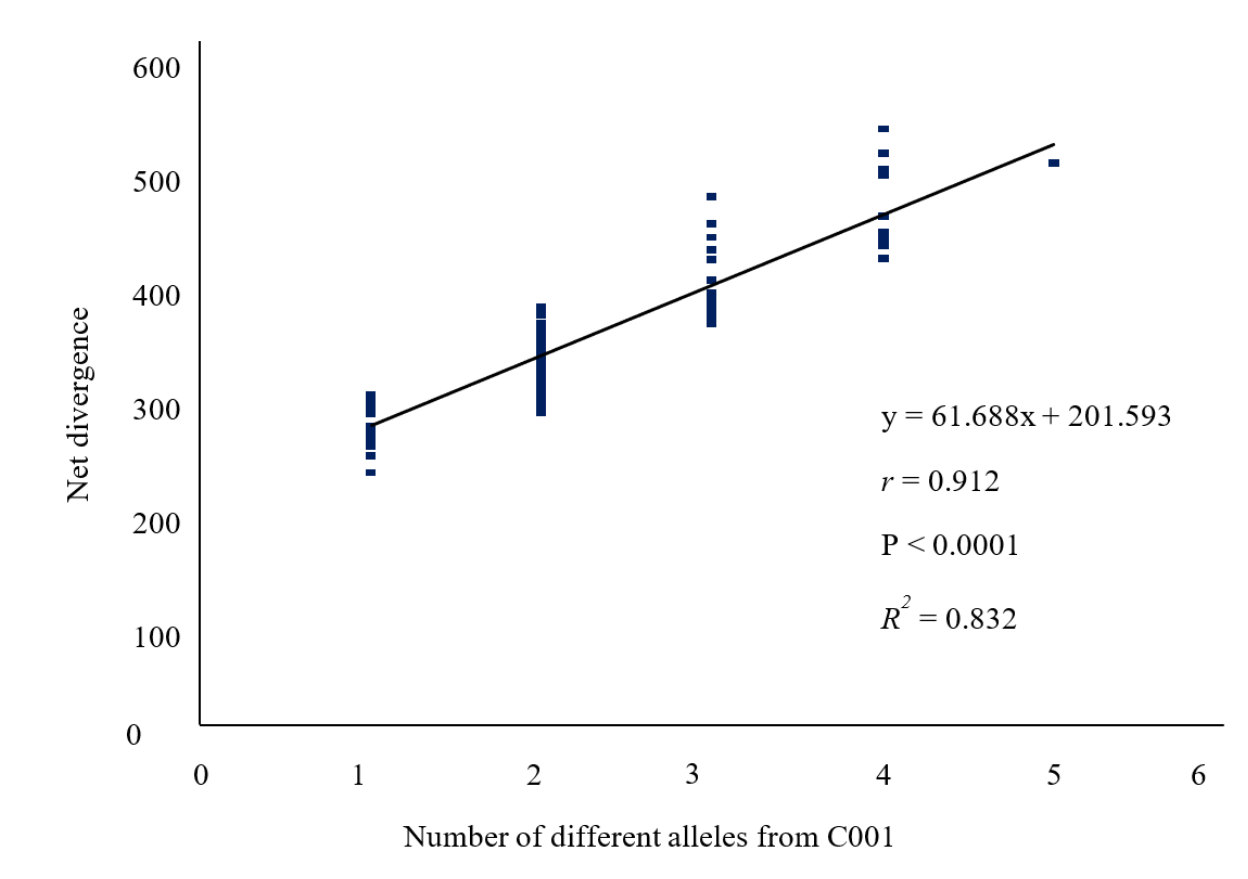

Supplement: Supplementary Figure 4 — Correlation between the number of different alleles of 94 ‘Galega vulgar’ genets in comparison to the most frequent genet (C001) and their net divergence. [file Image_4.JPEG]

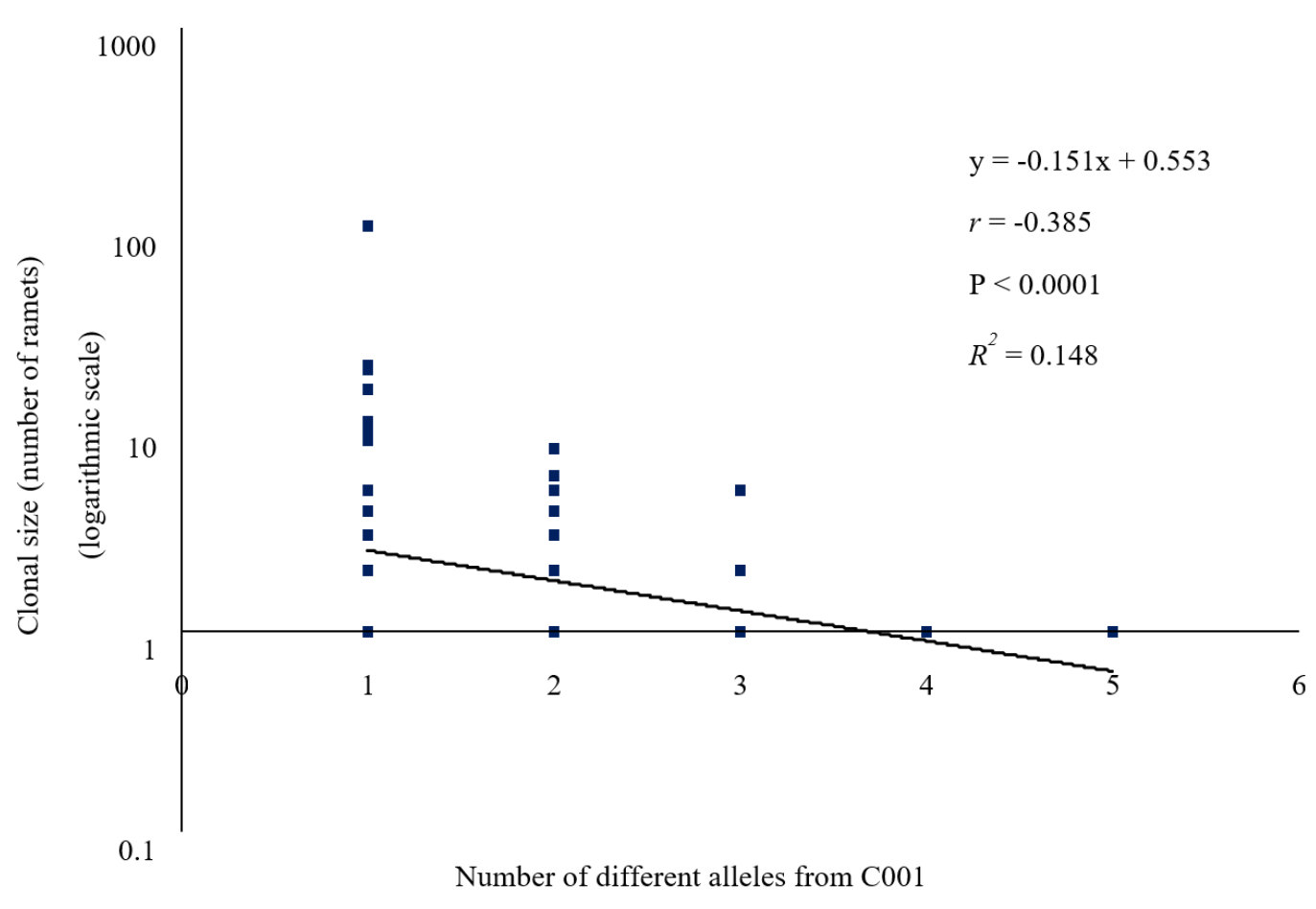

Supplement: Supplementary Figure 5 — Correlation between the number of different alleles of 94 ‘Galega vulgar’ genets in comparison to the most frequent genet (C001) and their genet size (number of ramets) (logarithmic scale). [file Image_5.JPEG]
