# Supplementary material for: Accessing Ancestral Origin and Diversity Evolution by Net Divergence of an Ongoing Domestication Mediterranean Olive Tree Variety
Source: Front Plant Sci. 2021 Jun 24;12:688214. doi: 10.3389/fpls.2021.688214 (PMC8265600; doi:10.3389/fpls.2021.688214)
Supplement: Supplementary Table 1 — Passport information of the 595 ‘Galega vulgar’ genotypes prospected and evaluated in this study, their endocarp profile and calculated age in years (according to Michelakis, 2002; Koniditsiotis, 2020). [file Data_Sheet_1.docx]

# Supplementary Tables

**Supplementary Table 1.** Passport information of the 595 ‘Galega vulgar’ genotypes prospected and evaluated in this study, their endocarp profile and calculated age in years (according to Michelakis (2002); Koniditsiotis (2020)).

| **Genotype ID** | **Genet** | **District** | **Latitude** | **Longitude** | **Germplasm group** | **Age group** | **Location** | **Endocarp Profile** |
| --- | --- | --- | --- | --- | --- | --- | --- | --- |
| agpb01 | C001 | Viseu | 40.23 | -8.14 | A | A1 | L2 | E1 |
| agpb03 | C001 | Viseu | 40.23 | -8.14 | A | A1 | L2 | E1 |
| agpb04 | C001 | Viseu | 40.23 | -8.14 | A | A1 | L2 | E1 |
| agpb05 | C037 | Viseu | 40.23 | -8.14 | A | A1 | L2 | E1 |
| ohlb11 | C021 | Viseu | 40.40 | -7.86 | A | A1 | L2 | E4 |
| ohlb12 | C001 | Viseu | 40.40 | -7.86 | A | A1 | L2 | E2 |
| flad01 | C001 | Viseu | 40.51 | -7.85 | C | C1 | L2 | E3 |
| flad02 | C001 | Viseu | 40.51 | -7.85 | C | C1 | L2 | E1 |
| llqt01 | C001 | Viseu | 40.47 | -7.92 | C | C1 | L1 | E1 |
| llqt02 | C003 | Viseu | 40.47 | -7.92 | C | C1 | L1 | E1 |
| llqt03 | C001 | Viseu | 40.47 | -7.92 | C | C1 | L1 | E2 |
| llqt04 | C001 | Viseu | 40.47 | -7.92 | C | C1 | L1 | E2 |
| llqt05 | C038 | Viseu | 40.47 | -7.92 | C | C1 | L1 | E2 |
| llqt06 | C001 | Viseu | 40.47 | -7.92 | C | C1 | L1 | E1 |
| llqt07 | C001 | Viseu | 40.47 | -7.92 | C | C1 | L1 | E1 |
| llqt08 | C001 | Viseu | 40.47 | -7.92 | C | C1 | L1 | E2 |
| llqt09 | C001 | Viseu | 40.47 | -7.92 | C | C1 | L1 | E1 |
| llqt10 | C001 | Viseu | 40.47 | -7.92 | C | C1 | L1 | E1 |
| nlqf01 | C003 | Viseu | 40.56 | -7.87 | C | C1 | L1 | E1 |
| nlqf02 | C001 | Viseu | 40.56 | -7.87 | C | C1 | L1 | E1 |
| nlqf03 | C001 | Viseu | 40.56 | -7.87 | C | C1 | L1 | E2 |
| nlqf04 | C001 | Viseu | 40.56 | -7.87 | C | C1 | L1 | E1 |
| nlqf05 | C008 | Viseu | 40.56 | -7.87 | C | C1 | L1 | E3 |
| nlqf06 | C001 | Viseu | 40.56 | -7.87 | C | C1 | L1 | E1 |
| nlqf07 | C001 | Viseu | 40.56 | -7.87 | C | C1 | L1 | E3 |
| nlqf08 | C001 | Viseu | 40.56 | -7.87 | C | C1 | L1 | E1 |
| nlqf09 | C001 | Viseu | 40.56 | -7.87 | C | C1 | L1 | E3 |
| nlqf10 | C003 | Viseu | 40.56 | -7.87 | C | C1 | L1 | E4 |
| nlqf11 | C001 | Viseu | 40.56 | -7.87 | C | C1 | L1 | E1 |
| nlqf12 | C020 | Viseu | 40.56 | -7.87 | C | C1 | L1 | E1 |
| nlqf13 | C039 | Viseu | 40.56 | -7.87 | C | C1 | L1 | E1 |
| nlqf14 | C003 | Viseu | 40.56 | -7.87 | C | C1 | L1 | E1 |
| ohbd01 | C001 | Viseu | 40.36 | -7.89 | C | C1 | L1 | E1 |
| ohbd02 | C007 | Viseu | 40.36 | -7.89 | C | C1 | L1 | E1 |
| ohbd03 | C001 | Viseu | 40.36 | -7.89 | C | C1 | L1 | E1 |
| ohbd04 | C001 | Viseu | 40.36 | -7.89 | C | C1 | L1 | E2 |
| ohbd05 | C040 | Viseu | 40.36 | -7.89 | C | C1 | L1 | E1 |
| ohbd06 | C007 | Viseu | 40.36 | -7.89 | C | C1 | L1 | E1 |
| ohbd07 | C007 | Viseu | 40.36 | -7.89 | C | C1 | L1 | E1 |
| ohbd08 | C007 | Viseu | 40.36 | -7.89 | C | C1 | L1 | E1 |
| ohbd09 | C001 | Viseu | 40.36 | -7.89 | C | C1 | L1 | E2 |
| ohbd10 | C001 | Viseu | 40.36 | -7.89 | C | C1 | L1 | E3 |
| ohaj01 | C001 | Viseu | 40.38 | -7.86 | C | C1 | L1 | E1 |
| ohaj02 | C019 | Viseu | 40.38 | -7.86 | C | C1 | L1 | E1 |
| ohaj03 | C001 | Viseu | 40.38 | -7.86 | C | C1 | L1 | E1 |
| ohaj04 | C001 | Viseu | 40.38 | -7.86 | C | C1 | L1 | E1 |
| ohaj05 | C001 | Viseu | 40.38 | -7.86 | C | C1 | L1 | E1 |
| ohaj06 | C019 | Viseu | 40.38 | -7.86 | C | C1 | L1 | E1 |
| ohaj07 | C008 | Viseu | 40.38 | -7.86 | C | C1 | L1 | E1 |
| ohaj08 | C019 | Viseu | 40.38 | -7.86 | C | C1 | L1 | E3 |
| ohaj09 | C001 | Viseu | 40.38 | -7.86 | C | C1 | L1 | E1 |
| ohaj10 | C008 | Viseu | 40.38 | -7.86 | C | C1 | L1 | E1 |
| ohlb01 | C001 | Viseu | 40.40 | -7.84 | C | C1 | L1 | E1 |
| ohlb02 | C001 | Viseu | 40.40 | -7.84 | C | C1 | L1 | E1 |
| ohlb03 | C001 | Viseu | 40.40 | -7.84 | C | C1 | L1 | E1 |
| ohlb04 | C008 | Viseu | 40.40 | -7.84 | C | C1 | L1 | E3 |
| ohlb05 | C001 | Viseu | 40.40 | -7.84 | C | C1 | L1 | E1 |
| ohlb06 | C001 | Viseu | 40.40 | -7.84 | C | C1 | L1 | E3 |
| ohlb07 | C001 | Viseu | 40.40 | -7.84 | C | C1 | L1 | E1 |
| ohlb08 | C001 | Viseu | 40.40 | -7.84 | C | C1 | L1 | E1 |
| ohlb09 | C004 | Viseu | 40.40 | -7.84 | C | C1 | L1 | E3 |
| ohlb10 | C004 | Viseu | 40.40 | -7.84 | C | C1 | L1 | E1 |
| ohsg01 | C005 | Viseu | 40.34 | -7.80 | C | C1 | L1 | E3 |
| ohsg02 | C001 | Viseu | 40.34 | -7.80 | C | C1 | L1 | E1 |
| ohsg03 | C005 | Viseu | 40.34 | -7.80 | C | C1 | L1 | E1 |
| ohsg04 | C011 | Viseu | 40.34 | -7.80 | C | C1 | L1 | E3 |
| ohsg05 | C041 | Viseu | 40.34 | -7.80 | C | C1 | L1 | E2 |
| ohsg06 | C005 | Viseu | 40.34 | -7.80 | C | C1 | L1 | E2 |
| ohsg07 | C005 | Viseu | 40.34 | -7.80 | C | C1 | L1 | E2 |
| ohsg08 | C001 | Viseu | 40.34 | -7.80 | C | C1 | L1 | E1 |
| ohsg09 | C005 | Viseu | 40.34 | -7.80 | C | C1 | L1 | E2 |
| ohsg10 | C006 | Viseu | 40.34 | -7.80 | C | C1 | L1 | E1 |
| tbtp01 | C001 | Viseu | 40.37 | -8.00 | C | C1 | L1 | E1 |
| tbtp02 | C001 | Viseu | 40.37 | -8.00 | C | C1 | L1 | E1 |
| tbtp03 | C011 | Viseu | 40.37 | -8.00 | C | C1 | L1 | E1 |
| tbtp04 | C001 | Viseu | 40.37 | -8.00 | C | C1 | L1 | E1 |
| tbtp05 | C001 | Viseu | 40.37 | -8.00 | C | C1 | L1 | E1 |
| tbtp06 | C025 | Viseu | 40.37 | -8.00 | C | C1 | L1 | E1 |
| tbtp07 | C001 | Viseu | 40.37 | -8.00 | C | C1 | L1 | E1 |
| tbtp08 | C007 | Viseu | 40.37 | -8.00 | C | C1 | L1 | E1 |
| tbtp09 | C001 | Viseu | 40.37 | -8.00 | C | C1 | L1 | E1 |
| tbtp10 | C001 | Viseu | 40.37 | -8.00 | C | C1 | L1 | E1 |
| cdcf01 | C001 | Coimbra | 40.10 | -8.50 | C | C1 | L1 | E3 |
| cdcf02 | C001 | Coimbra | 40.10 | -8.50 | C | C1 | L1 | E3 |
| cdcf03 | C002 | Coimbra | 40.10 | -8.50 | C | C1 | L1 | E1 |
| cdcf04 | C042 | Coimbra | 40.10 | -8.50 | C | C1 | L1 | E1 |
| cdcf05 | C001 | Coimbra | 40.10 | -8.50 | C | C1 | L1 | E3 |
| cdcf06 | C003 | Coimbra | 40.10 | -8.50 | C | C1 | L1 | E1 |
| cdcf07 | C001 | Coimbra | 40.10 | -8.50 | C | C1 | L1 | E1 |
| cdcf08 | C006 | Coimbra | 40.10 | -8.50 | C | C1 | L1 | E1 |
| cdcf09 | C001 | Coimbra | 40.10 | -8.50 | C | C1 | L1 | E1 |
| cdcf10 | C001 | Coimbra | 40.10 | -8.50 | C | C1 | L1 | E1 |
| bmln01 | C001 | Castelo Branco | 40.36 | -7.35 | A | A1 | L2 | E1 |
| vrsa01 | C002 | Castelo Branco | 39.65 | -7.67 | A | A2 | L2 | E3 |
| vrsa02 | C002 | Castelo Branco | 39.65 | -7.67 | A | A2 | L2 | E3 |
| vrsa03 | C001 | Castelo Branco | 39.65 | -7.67 | A | A2 | L2 | E1 |
| vrsa04 | C001 | Castelo Branco | 39.65 | -7.67 | A | A2 | L2 | E1 |
| vrsa05 | C002 | Castelo Branco | 39.65 | -7.67 | A | A2 | L2 | E1 |
| pnms01 | C001 | Castelo Branco | 39.78 | -7.81 | A | A3 | L2 | E3 |
| vrbf01 | C006 | Castelo Branco | 39.65 | -7.62 | A | A1 | L2 | E1 |
| vrrl01 | C001 | Castelo Branco | 39.67 | -7.61 | A | A1 | L2 | E1 |
| cbmb01 | C043 | Castelo Branco | 39.73 | -7.30 | C | C1 | L1 | E2 |
| cbmb02 | C002 | Castelo Branco | 39.73 | -7.30 | C | C1 | L1 | E2 |
| cbmb03 | C001 | Castelo Branco | 39.73 | -7.30 | C | C1 | L1 | E3 |
| cbmb04 | C001 | Castelo Branco | 39.73 | -7.30 | C | C1 | L1 | E3 |
| cbmb05 | C012 | Castelo Branco | 39.73 | -7.30 | C | C1 | L1 | E2 |
| cbmb06 | C002 | Castelo Branco | 39.73 | -7.30 | C | C1 | L1 | E2 |
| cbmb07 | C044 | Castelo Branco | 39.73 | -7.30 | C | C1 | L1 | E2 |
| cbmb08 | C024 | Castelo Branco | 39.73 | -7.30 | C | C1 | L1 | E4 |
| cbmb09 | C008 | Castelo Branco | 39.73 | -7.30 | C | C1 | L1 | E2 |
| cbmb10 | C001 | Castelo Branco | 39.73 | -7.30 | C | C1 | L1 | E2 |
| cbsz01 | C001 | Castelo Branco | 39.87 | -7.67 | C | C1 | L1 | E3 |
| cbsz02 | C001 | Castelo Branco | 39.87 | -7.67 | C | C1 | L1 | E1 |
| cbsz03 | C001 | Castelo Branco | 39.87 | -7.67 | C | C1 | L1 | E1 |
| cbsz04 | C001 | Castelo Branco | 39.87 | -7.67 | C | C1 | L1 | E1 |
| cbsz05 | C001 | Castelo Branco | 39.87 | -7.67 | C | C1 | L1 | E3 |
| cbsz06 | C001 | Castelo Branco | 39.87 | -7.67 | C | C1 | L1 | E3 |
| cbsz07 | C001 | Castelo Branco | 39.87 | -7.67 | C | C1 | L1 | E1 |
| cbsz08 | C001 | Castelo Branco | 39.87 | -7.67 | C | C1 | L1 | E1 |
| cbsz09 | C020 | Castelo Branco | 39.87 | -7.67 | C | C1 | L1 | E3 |
| cbsz10 | C045 | Castelo Branco | 39.87 | -7.68 | C | C1 | L1 | E1 |
| pvfd01 | C046 | Castelo Branco | 40.02 | -7.26 | C | C1 | L1 | E3 |
| pvfd02 | C002 | Castelo Branco | 40.02 | -7.26 | C | C1 | L1 | E1 |
| pvfd03 | C001 | Castelo Branco | 40.02 | -7.26 | C | C1 | L1 | E1 |
| pvfd04 | C002 | Castelo Branco | 40.04 | -7.27 | C | C1 | L1 | E1 |
| pvfd05 | C007 | Castelo Branco | 40.02 | -7.26 | C | C1 | L1 | E1 |
| pvfd06 | C001 | Castelo Branco | 40.02 | -7.26 | C | C1 | L1 | E1 |
| pvfd07 | C007 | Castelo Branco | 40.02 | -7.26 | C | C1 | L1 | E3 |
| pvfd08 | C007 | Castelo Branco | 40.02 | -7.26 | C | C1 | L1 | E3 |
| pvfd09 | C014 | Castelo Branco | 40.02 | -7.26 | C | C1 | L1 | E3 |
| semp02 | C002 | Castelo Branco | 39.88 | -6.92 | C | C1 | L1 | E1 |
| semp03 | C006 | Castelo Branco | 39.88 | -6.92 | C | C1 | L1 | E1 |
| semp04 | C002 | Castelo Branco | 39.88 | -6.92 | C | C1 | L1 | E1 |
| sete01 | C002 | Castelo Branco | 39.88 | -6.92 | C | C1 | L1 | E2 |
| sete02 | C047 | Castelo Branco | 39.88 | -6.92 | C | C1 | L1 | E3 |
| sete03 | C002 | Castelo Branco | 39.88 | -6.92 | C | C1 | L1 | E3 |
| sete04 | C007 | Castelo Branco | 39.88 | -6.92 | C | C1 | L1 | E1 |
| sete05 | C007 | Castelo Branco | 39.88 | -6.92 | C | C1 | L1 | E3 |
| pncr01 | C001 | Leiria | 39.93 | -8.41 | A | A2 | L1 | E4 |
| pncr02 | C001 | Leiria | 39.93 | -8.41 | A | A2 | L1 | E3 |
| pncr03 | C001 | Leiria | 39.93 | -8.41 | A | A2 | L1 | E3 |
| pncr04 | C001 | Leiria | 39.93 | -8.41 | A | A2 | L1 | E1 |
| pncr05 | C048 | Leiria | 39.93 | -8.41 | A | A2 | L1 | E2 |
| pncr06 | C002 | Leiria | 39.93 | -8.41 | A | A2 | L1 | E4 |
| pncr07 | C001 | Leiria | 39.93 | -8.41 | A | A2 | L1 | E4 |
| pncr08 | C001 | Leiria | 39.93 | -8.41 | A | A2 | L1 | E2 |
| pncr09 | C001 | Leiria | 39.93 | -8.41 | A | A2 | L1 | E1 |
| pncr10 | C002 | Leiria | 39.93 | -8.41 | A | A2 | L1 | E1 |
| pncr11 | C028 | Leiria | 39.93 | -8.41 | A | A2 | L1 | E4 |
| pncr12 | C011 | Leiria | 39.93 | -8.41 | A | A2 | L1 | E1 |
| pncr13 | C001 | Leiria | 39.93 | -8.41 | A | A2 | L1 | E1 |
| pncr14 | C001 | Leiria | 39.93 | -8.41 | A | A2 | L1 | E1 |
| pncr15 | C001 | Leiria | 39.93 | -8.40 | A | A2 | L1 | E4 |
| pbrd01 | C015 | Leiria | 40.00 | -8.58 | A | A1 | L2 | E3 |
| pbrd02 | C001 | Leiria | 39.98 | -8.58 | A | A1 | L2 | E1 |
| pbrd03 | C049 | Leiria | 39.98 | -8.58 | A | A1 | L2 | E3 |
| pbrd04 | C001 | Leiria | 39.95 | -8.56 | A | A1 | L2 | E4 |
| pbrd05 | C001 | Leiria | 39.95 | -8.55 | A | A1 | L2 | E1 |
| pnft01 | C001 | Leiria | 40.02 | -8.44 | A | A2 | L2 | E4 |
| pnft02 | C001 | Leiria | 40.02 | -8.44 | A | A2 | L2 | E4 |
| pnft03 | C001 | Leiria | 40.02 | -8.44 | A | A2 | L2 | E4 |
| pnft04 | C001 | Leiria | 40.02 | -8.44 | A | A2 | L2 | E2 |
| pnvr01 | C001 | Leiria | 40.04 | -8.46 | A | A1 | L1 | E4 |
| pnvr02 | C001 | Leiria | 40.04 | -8.46 | A | A1 | L1 | E1 |
| pnvr03 | C001 | Leiria | 40.04 | -8.46 | A | A1 | L1 | E2 |
| anra01 | C001 | Leiria | 40.01 | -8.46 | C | C1 | L1 | E1 |
| anra02 | C001 | Leiria | 40.01 | -8.46 | C | C1 | L1 | E3 |
| anra03 | C001 | Leiria | 40.01 | -8.46 | C | C1 | L1 | E1 |
| anra04 | C001 | Leiria | 40.01 | -8.46 | C | C1 | L1 | E2 |
| anra05 | C001 | Leiria | 40.01 | -8.46 | C | C1 | L1 | E1 |
| anra06 | C015 | Leiria | 40.01 | -8.46 | C | C1 | L1 | E3 |
| anra07 | C001 | Leiria | 40.01 | -8.46 | C | C1 | L1 | E1 |
| anra08 | C001 | Leiria | 40.01 | -8.46 | C | C1 | L1 | E1 |
| anra09 | C001 | Leiria | 40.01 | -8.46 | C | C1 | L1 | E3 |
| lfam01 | C002 | Leiria | 39.60 | -8.64 | C | C1 | L1 | E2 |
| lfam02 | C002 | Leiria | 39.60 | -8.64 | C | C1 | L1 | E4 |
| lfam03 | C001 | Leiria | 39.60 | -8.64 | C | C1 | L1 | E4 |
| lfam04 | C002 | Leiria | 39.60 | -8.64 | C | C1 | L1 | E1 |
| lfam05 | C002 | Leiria | 39.60 | -8.64 | C | C1 | L1 | E1 |
| lfam06 | C026 | Leiria | 39.60 | -8.64 | C | C1 | L1 | E3 |
| lfam07 | C002 | Leiria | 39.60 | -8.64 | C | C1 | L1 | E2 |
| lfam08 | C002 | Leiria | 39.60 | -8.64 | C | C1 | L1 | E3 |
| lfam09 | C026 | Leiria | 39.60 | -8.64 | C | C1 | L1 | E1 |
| lfam10 | C001 | Leiria | 39.60 | -8.64 | C | C1 | L1 | E3 |
| lfmnt01 | C018 | Leiria | 39.60 | -8.65 | C | C1 | L2 | E1 |
| lfco01 | C050 | Leiria | 39.61 | -8.65 | C | C1 | L2 | E2 |
| lfco02 | C001 | Leiria | 39.61 | -8.65 | C | C1 | L2 | E2 |
| arac01 | C016 | Portalegre | 39.11 | -7.29 | A | A2 | L1 | E1 |
| arac02 | C002 | Portalegre | 39.11 | -7.29 | A | A2 | L1 | E1 |
| arac03 | C002 | Portalegre | 39.11 | -7.29 | A | A2 | L1 | E3 |
| arac04 | C051 | Portalegre | 39.11 | -7.29 | A | A2 | L1 | E1 |
| arac05 | C002 | Portalegre | 39.11 | -7.29 | A | A2 | L1 | E1 |
| arac06 | C002 | Portalegre | 39.11 | -7.29 | A | A2 | L1 | E1 |
| arac07 | C002 | Portalegre | 39.11 | -7.29 | A | A2 | L1 | E1 |
| arac08 | C002 | Portalegre | 39.11 | -7.29 | A | A2 | L1 | E1 |
| arac09 | C002 | Portalegre | 39.12 | -7.29 | A | A2 | L1 | E1 |
| arac10 | C012 | Portalegre | 39.12 | -7.29 | A | A2 | L1 | E1 |
| arac11 | C002 | Portalegre | 39.11 | -7.29 | A | A2 | L1 | E1 |
| arac12 | C002 | Portalegre | 39.11 | -7.29 | A | A2 | L1 | E1 |
| arac13 | C002 | Portalegre | 39.11 | -7.29 | A | A2 | L1 | E3 |
| arac14 | C002 | Portalegre | 39.11 | -7.29 | A | A2 | L1 | E1 |
| arac15 | C002 | Portalegre | 39.11 | -7.29 | A | A2 | L1 | E1 |
| arac16 | C002 | Portalegre | 39.11 | -7.29 | A | A2 | L1 | E1 |
| arac17 | C020 | Portalegre | 39.11 | -7.29 | A | A2 | L1 | E1 |
| arac18 | C002 | Portalegre | 39.11 | -7.29 | A | A2 | L1 | E3 |
| arac19 | C002 | Portalegre | 39.11 | -7.29 | A | A2 | L1 | E1 |
| arac20 | C002 | Portalegre | 39.11 | -7.29 | A | A2 | L1 | E1 |
| avmc01 | C052 | Portalegre | 39.07 | -7.88 | C | C1 | L1 | E2 |
| avmc02 | C033 | Portalegre | 39.07 | -7.88 | C | C1 | L1 | E2 |
| avmc03 | C001 | Portalegre | 39.07 | -7.88 | C | C1 | L1 | E1 |
| avmc04 | C001 | Portalegre | 39.07 | -7.88 | C | C1 | L1 | E4 |
| avmc05 | C001 | Portalegre | 39.07 | -7.88 | C | C1 | L1 | E2 |
| avmc06 | C002 | Portalegre | 39.07 | -7.88 | C | C1 | L1 | E2 |
| avmc07 | C001 | Portalegre | 39.07 | -7.88 | C | C1 | L1 | E2 |
| avmc08 | C010 | Portalegre | 39.07 | -7.88 | C | C1 | L1 | E2 |
| avmc09 | C001 | Portalegre | 39.07 | -7.88 | C | C1 | L1 | E4 |
| avmc10 | C001 | Portalegre | 39.07 | -7.88 | C | C1 | L1 | E2 |
| elvb01 | C002 | Portalegre | 38.87 | -7.27 | C | C1 | L1 | E3 |
| elvb02 | C018 | Portalegre | 38.87 | -7.27 | C | C1 | L1 | E1 |
| elvb03 | C027 | Portalegre | 38.87 | -7.27 | C | C1 | L1 | E1 |
| elvb05 | C053 | Portalegre | 38.87 | -7.27 | C | C1 | L1 | E1 |
| elvb06 | C001 | Portalegre | 38.87 | -7.27 | C | C1 | L1 | E3 |
| elvb07 | C002 | Portalegre | 38.87 | -7.27 | C | C1 | L1 | E1 |
| elvb08 | C002 | Portalegre | 38.87 | -7.27 | C | C1 | L1 | E1 |
| elvb09 | C015 | Portalegre | 38.87 | -7.27 | C | C1 | L1 | E2 |
| elvb10 | C054 | Portalegre | 38.87 | -7.27 | C | C1 | L1 | E2 |
| mvmn03 | C005 | Portalegre | 39.37 | -7.33 | C | C1 | L1 | E2 |
| mvmn05 | C055 | Portalegre | 39.37 | -7.33 | C | C1 | L1 | E1 |
| mvmn06 | C001 | Portalegre | 39.37 | -7.33 | C | C1 | L1 | E1 |
| mvmn07 | C001 | Portalegre | 39.37 | -7.33 | C | C1 | L1 | E1 |
| mvmn08 | C001 | Portalegre | 39.37 | -7.33 | C | C1 | L1 | E1 |
| mvmn09 | C002 | Portalegre | 39.37 | -7.33 | C | C1 | L1 | E1 |
| mvmn10 | C056 | Portalegre | 39.37 | -7.33 | C | C1 | L1 | E1 |
| ptmv01 | C001 | Portalegre | 39.28 | -7.40 | C | C1 | L1 | E1 |
| ptmv02 | C057 | Portalegre | 39.28 | -7.40 | C | C1 | L1 | E1 |
| ptmv03 | C001 | Portalegre | 39.28 | -7.40 | C | C1 | L1 | E2 |
| ptmv04 | C005 | Portalegre | 39.28 | -7.40 | C | C1 | L1 | E1 |
| ptmv05 | C001 | Portalegre | 39.28 | -7.40 | C | C1 | L1 | E2 |
| ptmv06 | C058 | Portalegre | 39.28 | -7.40 | C | C1 | L1 | E1 |
| ptmv07 | C016 | Portalegre | 39.28 | -7.40 | C | C1 | L1 | E1 |
| ptmv08 | C016 | Portalegre | 39.28 | -7.40 | C | C1 | L1 | E1 |
| ptmv09 | C005 | Portalegre | 39.28 | -7.40 | C | C1 | L1 | E1 |
| ptmv10 | C016 | Portalegre | 39.28 | -7.40 | C | C1 | L1 | E1 |
| sdvl01 | C004 | Santarém | 39.51 | -8.15 | A | A2 | L1 | E1 |
| sdvl02 | C002 | Santarém | 39.51 | -8.15 | A | A2 | L1 | E3 |
| sdvl03 | C001 | Santarém | 39.51 | -8.15 | A | A2 | L1 | E1 |
| sdvl04 | C004 | Santarém | 39.51 | -8.15 | A | A2 | L1 | E1 |
| sdvl05 | C001 | Santarém | 39.51 | -8.15 | A | A2 | L1 | E2 |
| sdvl06 | C002 | Santarém | 39.51 | -8.15 | A | A2 | L1 | E3 |
| sdvl07 | C001 | Santarém | 39.51 | -8.15 | A | A2 | L1 | E1 |
| sdvl08 | C002 | Santarém | 39.51 | -8.15 | A | A2 | L1 | E1 |
| sdvl09 | C003 | Santarém | 39.51 | -8.15 | A | A2 | L1 | E3 |
| sdvl10 | C004 | Santarém | 39.51 | -8.15 | A | A2 | L1 | E1 |
| sdvl11 | C001 | Santarém | 39.51 | -8.15 | A | A2 | L1 | E1 |
| sdvl12 | C059 | Santarém | 39.51 | -8.15 | A | A2 | L1 | E3 |
| ctqp11 | C017 | Santarém | 39.48 | -8.32 | A | A1 | L1 | E1 |
| ctqp12 | C001 | Santarém | 39.48 | -8.32 | A | A1 | L1 | E2 |
| ctqp13 | C001 | Santarém | 39.48 | -8.32 | A | A1 | L1 | E1 |
| ctqp14 | C002 | Santarém | 39.48 | -8.32 | A | A1 | L1 | E1 |
| ctqp15 | C001 | Santarém | 39.48 | -8.32 | A | A1 | L1 | E1 |
| ctqp16 | C060 | Santarém | 39.48 | -8.32 | A | A1 | L1 | E3 |
| ctqp17 | C002 | Santarém | 39.48 | -8.32 | A | A1 | L1 | E3 |
| ctqp18 | C002 | Santarém | 39.48 | -8.32 | A | A1 | L1 | E3 |
| ctqp19 | C061 | Santarém | 39.48 | -8.32 | A | A1 | L1 | E1 |
| ctqp20 | C001 | Santarém | 39.48 | -8.32 | A | A1 | L1 | E1 |
| sdfn01 | C001 | Santarém | 39.53 | -8.17 | A | A2 | L1 | E3 |
| sdfn02 | C001 | Santarém | 39.53 | -8.17 | A | A2 | L1 | E3 |
| sdfn03 | C001 | Santarém | 39.53 | -8.17 | A | A2 | L1 | E3 |
| sdfn04 | C001 | Santarém | 39.53 | -8.17 | A | A2 | L1 | E1 |
| sdfn05 | C011 | Santarém | 39.53 | -8.17 | A | A2 | L1 | E3 |
| sdfn06 | C001 | Santarém | 39.53 | -8.17 | A | A2 | L1 | E1 |
| sdfn07 | C062 | Santarém | 39.53 | -8.17 | A | A2 | L1 | E3 |
| sdfn08 | C001 | Santarém | 39.53 | -8.17 | A | A2 | L1 | E2 |
| sdfn09 | C001 | Santarém | 39.53 | -8.17 | A | A2 | L1 | E3 |
| sdfn10 | C004 | Santarém | 39.53 | -8.17 | A | A2 | L1 | E2 |
| sdsm11 | C029 | Santarém | 39.51 | -8.15 | A | A1 | L1 | E1 |
| sdsm12 | C013 | Santarém | 39.51 | -8.15 | A | A1 | L1 | E3 |
| sdsm13 | C009 | Santarém | 39.47 | -8.14 | A | A1 | L1 | E1 |
| sdsm14 | C001 | Santarém | 39.51 | -8.15 | A | A1 | L1 | E3 |
| sdsm15 | C001 | Santarém | 39.51 | -8.15 | A | A1 | L1 | E1 |
| sdsm16 | C009 | Santarém | 39.51 | -8.15 | A | A1 | L1 | E1 |
| sdsm17 | C002 | Santarém | 39.51 | -8.15 | A | A1 | L1 | E3 |
| sdsm18 | C001 | Santarém | 39.51 | -8.15 | A | A1 | L1 | E3 |
| sdsm19 | C063 | Santarém | 39.51 | -8.15 | A | A1 | L1 | E1 |
| sdsm20 | C001 | Santarém | 39.51 | -8.15 | A | A1 | L1 | E1 |
| mceo01 | C002 | Santarém | 39.47 | -8.04 | A | A2 | L1 | E1 |
| mceo02 | C011 | Santarém | 39.47 | -8.04 | A | A2 | L1 | E2 |
| mceo03 | C001 | Santarém | 39.47 | -8.04 | A | A2 | L1 | E1 |
| mceo04 | C004 | Santarém | 39.47 | -8.04 | A | A2 | L1 | E1 |
| mceo05 | C001 | Santarém | 39.47 | -8.04 | A | A2 | L1 | E1 |
| mceo06 | C001 | Santarém | 39.47 | -8.04 | A | A2 | L1 | E1 |
| mceo07 | C001 | Santarém | 39.47 | -8.03 | A | A2 | L1 | E1 |
| ctes01 | C002 | Santarém | 39.48 | -8.32 | A | A2 | L1 | E1 |
| ctes02 | C017 | Santarém | 39.48 | -8.32 | A | A2 | L1 | E2 |
| ctes03 | C002 | Santarém | 39.48 | -8.33 | A | A2 | L1 | E1 |
| ctes04 | C064 | Santarém | 39.48 | -8.33 | A | A2 | L1 | E1 |
| ctes05 | C028 | Santarém | 39.48 | -8.33 | A | A2 | L1 | E2 |
| sdtl01 | C004 | Santarém | 39.53 | -8.17 | A | A2 | L1 | E3 |
| sdtl02 | C029 | Santarém | 39.53 | -8.17 | A | A2 | L1 | E1 |
| sdtl03 | C001 | Santarém | 39.53 | -8.17 | A | A2 | L1 | E1 |
| sdtl04 | C001 | Santarém | 39.53 | -8.17 | A | A2 | L1 | E1 |
| sdtl05 | C001 | Santarém | 39.53 | -8.17 | A | A2 | L1 | E3 |
| mcet01 | C001 | Santarém | 39.51 | -8.04 | A | A1 | L1 | E3 |
| mcet02 | C001 | Santarém | 39.51 | -8.04 | A | A1 | L1 | E1 |
| mcet03 | C001 | Santarém | 39.50 | -8.04 | A | A1 | L1 | E3 |
| mcom01 | C002 | Santarém | 39.47 | -8.08 | A | A3 | L2 | E1 |
| stsb01 | C022 | Santarém | 39.31 | -8.72 | A | A1 | L2 | E3 |
| stsb02 | C001 | Santarém | 39.31 | -8.72 | A | A3 | L2 | E1 |
| cdre01 | C021 | Santarém | 39.24 | -9.10 | A | A1 | L2 | E1 |
| azac01 | C017 | Santarém | 39.14 | -8.90 | A | A3 | L2 | E1 |
| azsm01 | C031 | Santarém | 39.08 | -8.88 | A | A2 | L2 | E3 |
| vbqp01 | C004 | Santarém | 39.48 | -8.43 | A | A1 | L2 | E1 |
| vbqq01 | C065 | Santarém | 39.46 | -8.45 | A | A2 | L1 | E1 |
| vbqq02 | C001 | Santarém | 39.46 | -8.45 | A | A2 | L1 | E3 |
| vbqq03 | C003 | Santarém | 39.46 | -8.45 | A | A2 | L1 | E1 |
| vbqq04 | C066 | Santarém | 39.46 | -8.45 | A | A2 | L1 | E1 |
| vbqq05 | C022 | Santarém | 39.46 | -8.45 | A | A2 | L1 | E1 |
| vbqq06 | C067 | Santarém | 39.46 | -8.45 | A | A2 | L1 | E1 |
| vbqq07 | C005 | Santarém | 39.46 | -8.45 | A | A2 | L1 | E1 |
| vbqq08 | C001 | Santarém | 39.46 | -8.45 | A | A2 | L1 | E1 |
| vbqq09 | C001 | Santarém | 39.46 | -8.45 | A | A2 | L1 | E1 |
| vbqq10 | C022 | Santarém | 39.46 | -8.45 | A | A2 | L1 | E3 |
| vbqq11 | C001 | Santarém | 39.46 | -8.45 | A | A2 | L1 | E3 |
| vbqq12 | C001 | Santarém | 39.46 | -8.45 | A | A2 | L1 | E1 |
| tnar01 | C005 | Santarém | 39.52 | -8.54 | A | A2 | L2 | E1 |
| tnar02 | C001 | Santarém | 39.52 | -8.54 | A | A2 | L2 | E1 |
| tnar03 | C001 | Santarém | 39.52 | -8.55 | A | A2 | L2 | E1 |
| tncp01 | C003 | Santarém | 39.46 | -8.56 | A | A2 | L2 | E1 |
| tncp02 | C001 | Santarém | 39.46 | -8.56 | A | A2 | L2 | E3 |
| tncp04 | C068 | Santarém | 39.46 | -8.56 | A | A2 | L2 | E3 |
| tnol01 | C001 | Santarém | 39.52 | -8.47 | A | A2 | L2 | E1 |
| abbp01 | C004 | Santarém | 39.34 | -8.09 | C | C1 | L1 | E3 |
| abbp02 | C001 | Santarém | 39.34 | -8.09 | C | C1 | L1 | E1 |
| abbp03 | C002 | Santarém | 39.34 | -8.09 | C | C1 | L1 | E1 |
| abbp04 | C004 | Santarém | 39.34 | -8.09 | C | C1 | L1 | E1 |
| abbp05 | C001 | Santarém | 39.34 | -8.09 | C | C1 | L1 | E1 |
| abbp06 | C004 | Santarém | 39.34 | -8.09 | C | C1 | L1 | E1 |
| abbp07 | C004 | Santarém | 39.34 | -8.09 | C | C1 | L1 | E1 |
| abbp08 | C002 | Santarém | 39.34 | -8.09 | C | C1 | L1 | E1 |
| abbp09 | C004 | Santarém | 39.34 | -8.09 | C | C1 | L1 | E1 |
| abbp10 | C004 | Santarém | 39.34 | -8.09 | C | C1 | L1 | E3 |
| abhl01 | C069 | Santarém | 39.48 | -8.13 | C | C1 | L1 | E1 |
| abhl02 | C001 | Santarém | 39.48 | -8.13 | C | C1 | L1 | E3 |
| abhl03 | C001 | Santarém | 39.48 | -8.13 | C | C1 | L1 | E3 |
| abhl04 | C030 | Santarém | 39.49 | -8.12 | C | C1 | L1 | E1 |
| abhl05 | C070 | Santarém | 39.49 | -8.12 | C | C1 | L1 | E1 |
| abhl06 | C001 | Santarém | 39.49 | -8.12 | C | C1 | L1 | E1 |
| abhl07 | C001 | Santarém | 39.49 | -8.12 | C | C1 | L1 | E1 |
| abhl08 | C001 | Santarém | 39.49 | -8.12 | C | C1 | L1 | E1 |
| abhl09 | C002 | Santarém | 39.49 | -8.12 | C | C1 | L1 | E1 |
| abhl10 | C071 | Santarém | 39.49 | -8.12 | C | C1 | L1 | E1 |
| absd01 | C009 | Santarém | 39.55 | -8.17 | C | C1 | L1 | E3 |
| absd02 | C009 | Santarém | 39.55 | -8.17 | C | C1 | L1 | E1 |
| absd03 | C009 | Santarém | 39.55 | -8.17 | C | C1 | L1 | E1 |
| absd04 | C002 | Santarém | 39.55 | -8.17 | C | C1 | L1 | E1 |
| absd05 | C001 | Santarém | 39.55 | -8.17 | C | C1 | L1 | E3 |
| absd06 | C009 | Santarém | 39.55 | -8.17 | C | C1 | L1 | E1 |
| absd07 | C004 | Santarém | 39.55 | -8.17 | C | C1 | L1 | E1 |
| absd08 | C001 | Santarém | 39.55 | -8.17 | C | C1 | L1 | E3 |
| absd09 | C002 | Santarém | 39.55 | -8.17 | C | C1 | L1 | E3 |
| absd10 | C004 | Santarém | 39.55 | -8.17 | C | C1 | L1 | E1 |
| ctqp01 | C072 | Santarém | 39.48 | -8.32 | C | C1 | L1 | E1 |
| ctqp02 | C002 | Santarém | 39.48 | -8.32 | C | C1 | L1 | E3 |
| ctqp03 | C001 | Santarém | 39.48 | -8.32 | C | C1 | L1 | E1 |
| ctqp04 | C025 | Santarém | 39.48 | -8.32 | C | C1 | L1 | E2 |
| ctqp05 | C001 | Santarém | 39.48 | -8.32 | C | C1 | L1 | E2 |
| ctqp08 | C001 | Santarém | 39.48 | -8.32 | C | C1 | L1 | E1 |
| ctqp09 | C002 | Santarém | 39.48 | -8.32 | C | C1 | L1 | E1 |
| ctsm01 | C001 | Santarém | 39.46 | -8.31 | C | C1 | L1 | E1 |
| ctsm02 | C001 | Santarém | 39.46 | -8.31 | C | C1 | L1 | E1 |
| ctsm03 | C018 | Santarém | 39.46 | -8.31 | C | C1 | L1 | E1 |
| ctsm04 | C001 | Santarém | 39.46 | -8.31 | C | C1 | L1 | E3 |
| ctsm05 | C001 | Santarém | 39.46 | -8.31 | C | C1 | L1 | E1 |
| ctsm06 | C002 | Santarém | 39.46 | -8.31 | C | C1 | L1 | E1 |
| ctsm07 | C002 | Santarém | 39.46 | -8.31 | C | C1 | L1 | E1 |
| ctsm08 | C002 | Santarém | 39.46 | -8.31 | C | C1 | L1 | E1 |
| ctsm09 | C001 | Santarém | 39.46 | -8.31 | C | C1 | L1 | E3 |
| ctsm10 | C002 | Santarém | 39.46 | -8.31 | C | C1 | L1 | E1 |
| rtog01 | C002 | Santarém | 39.43 | -8.22 | C | C1 | L1 | E3 |
| rtog02 | C003 | Santarém | 39.43 | -8.22 | C | C1 | L1 | E1 |
| rtog03 | C023 | Santarém | 39.43 | -8.22 | C | C1 | L1 | E1 |
| rtog04 | C002 | Santarém | 39.43 | -8.22 | C | C1 | L1 | E1 |
| rtog05 | C003 | Santarém | 39.43 | -8.22 | C | C1 | L1 | E1 |
| rtog06 | C002 | Santarém | 39.44 | -8.22 | C | C1 | L1 | E1 |
| rtog07 | C002 | Santarém | 39.43 | -8.22 | C | C1 | L1 | E3 |
| rtog08 | C006 | Santarém | 39.44 | -8.22 | C | C1 | L1 | E1 |
| sdsm02 | C013 | Santarém | 39.51 | -8.15 | C | C1 | L1 | E1 |
| sdsm06 | C001 | Santarém | 39.51 | -8.15 | C | C1 | L1 | E1 |
| sdsm07 | C009 | Santarém | 39.51 | -8.15 | C | C1 | L1 | E1 |
| sdsm08 | C001 | Santarém | 39.51 | -8.15 | C | C1 | L1 | E3 |
| sdsm09 | C004 | Santarém | 39.51 | -8.15 | C | C1 | L1 | E2 |
| sdsm10 | C013 | Santarém | 39.51 | -8.15 | C | C1 | L1 | E3 |
| vlsq01 | C014 | Santarém | 39.51 | -8.15 | C | C1 | L1 | E1 |
| vlsq02 | C004 | Santarém | 39.51 | -8.15 | C | C1 | L1 | E3 |
| vlsq03 | C006 | Santarém | 39.51 | -8.15 | C | C1 | L1 | E1 |
| vlsq04 | C001 | Santarém | 39.51 | -8.15 | C | C1 | L1 | E1 |
| vlsq05 | C001 | Santarém | 39.51 | -8.15 | C | C1 | L1 | E3 |
| vlsq06 | C008 | Santarém | 39.51 | -8.15 | C | C1 | L1 | E3 |
| vlsq07 | C002 | Santarém | 39.51 | -8.15 | C | C1 | L1 | E1 |
| vlsq08 | C001 | Santarém | 39.51 | -8.15 | C | C1 | L1 | E1 |
| vlsq09 | C001 | Santarém | 39.51 | -8.15 | C | C1 | L1 | E1 |
| vlsq10 | C009 | Santarém | 39.51 | -8.15 | C | C1 | L1 | E1 |
| lfmt01 | C002 | Santarém | 39.53 | -8.55 | C | C1 | L1 | E3 |
| lfmt02 | C002 | Santarém | 39.53 | -8.55 | C | C1 | L1 | E1 |
| lfmt03 | C018 | Santarém | 39.53 | -8.55 | C | C1 | L1 | E1 |
| lfmt04 | C073 | Santarém | 39.53 | -8.55 | C | C1 | L1 | E3 |
| lfmt05 | C002 | Santarém | 39.54 | -8.55 | C | C1 | L1 | E1 |
| lfmt06 | C001 | Santarém | 39.54 | -8.55 | C | C1 | L1 | E1 |
| lfmt07 | C074 | Santarém | 39.54 | -8.55 | C | C1 | L1 | E1 |
| lfmt08 | C002 | Santarém | 39.54 | -8.55 | C | C1 | L1 | E1 |
| lfmt09 | C002 | Santarém | 39.54 | -8.55 | C | C1 | L1 | E1 |
| lfmt10 | C002 | Santarém | 39.54 | -8.55 | C | C1 | L1 | E1 |
| lrhm01 | C002 | Lisboa | 38.80 | -9.10 | A | A3 | L2 | E1 |
| lrsa01 | C001 | Lisboa | 38.84 | -9.09 | A | A3 | L2 | E1 |
| alsa01 | C001 | Lisboa | 38.70 | -9.18 | A | A1 | L2 | E2 |
| alsa02 | C003 | Lisboa | 38.70 | -9.18 | A | A1 | L2 | E1 |
| alsa03 | C003 | Lisboa | 38.70 | -9.18 | A | A1 | L2 | E2 |
| alsa04 | C003 | Lisboa | 38.70 | -9.18 | A | A1 | L2 | E1 |
| alqb01 | C001 | Lisboa | 39.12 | -9.02 | A | A1 | L1 | E3 |
| alqb02 | C014 | Lisboa | 39.12 | -9.02 | A | A1 | L1 | E1 |
| alqb03 | C002 | Lisboa | 39.12 | -9.02 | A | A1 | L1 | E1 |
| alqb04 | C024 | Lisboa | 39.12 | -9.02 | A | A1 | L1 | E4 |
| alqb05 | C003 | Lisboa | 39.12 | -9.02 | A | A1 | L1 | E1 |
| alqb06 | C001 | Lisboa | 39.12 | -9.02 | A | A1 | L1 | E1 |
| alqb07 | C035 | Lisboa | 39.12 | -9.02 | A | A1 | L1 | E2 |
| alqb08 | C001 | Lisboa | 39.12 | -9.02 | A | A1 | L1 | E1 |
| alqb09 | C002 | Lisboa | 39.12 | -9.02 | A | A1 | L1 | E4 |
| alqb10 | C001 | Lisboa | 39.12 | -9.02 | A | A1 | L1 | E3 |
| alog01 | C075 | Lisboa | 39.11 | -8.99 | A | A2 | L1 | E3 |
| alog02 | C001 | Lisboa | 39.11 | -8.99 | A | A2 | L1 | E1 |
| alog03 | C001 | Lisboa | 39.11 | -8.99 | A | A2 | L1 | E1 |
| alog04 | C003 | Lisboa | 39.11 | -8.99 | A | A2 | L1 | E4 |
| alch01 | C031 | Lisboa | 39.07 | -8.99 | A | A1 | L2 | E3 |
| alch02 | C017 | Lisboa | 39.08 | -8.99 | A | A1 | L2 | E1 |
| alch03 | C076 | Lisboa | 39.08 | -8.99 | A | A1 | L2 | E1 |
| alsg01 | C002 | Lisboa | 39.14 | -9.02 | A | A1 | L2 | E4 |
| alsg02 | C012 | Lisboa | 39.14 | -9.02 | A | A1 | L2 | E3 |
| alsg03 | C003 | Lisboa | 39.14 | -9.02 | A | A1 | L2 | E1 |
| alot01 | C006 | Lisboa | 39.11 | -8.99 | A | A1 | L2 | E4 |
| alqg01 | C001 | Lisboa | 39.11 | -9.00 | A | A1 | L2 | E2 |
| azqq01 | C001 | Setúbal | 38.52 | -9.00 | A | A2 | L1 | E1 |
| azqq02 | C001 | Setúbal | 38.52 | -9.00 | A | A2 | L1 | E1 |
| azqq03 | C077 | Setúbal | 38.52 | -9.00 | A | A2 | L1 | E3 |
| azqq04 | C001 | Setúbal | 38.52 | -9.00 | A | A2 | L1 | E3 |
| azqq06 | C001 | Setúbal | 38.52 | -9.00 | A | A2 | L1 | E3 |
| azqq07 | C001 | Setúbal | 38.52 | -9.00 | A | A2 | L1 | E3 |
| azqq08 | C001 | Setúbal | 38.52 | -9.00 | A | A2 | L1 | E3 |
| azqq09 | C001 | Setúbal | 38.52 | -9.00 | A | A2 | L1 | E3 |
| azqq10 | C001 | Setúbal | 38.52 | -9.00 | A | A2 | L1 | E3 |
| azqq11 | C001 | Setúbal | 38.52 | -9.00 | A | A2 | L1 | E1 |
| azqq12 | C001 | Setúbal | 38.52 | -9.00 | A | A2 | L1 | E3 |
| azqq13 | C001 | Setúbal | 38.52 | -9.00 | A | A2 | L1 | E3 |
| azqq14 | C078 | Setúbal | 38.52 | -9.00 | A | A2 | L1 | E1 |
| azqq15 | C001 | Setúbal | 38.52 | -9.00 | A | A2 | L1 | E1 |
| azqq16 | C001 | Setúbal | 38.52 | -9.00 | A | A2 | L1 | E3 |
| azen01 | C024 | Setúbal | 38.52 | -9.00 | A | A3 | L2 | E4 |
| azen02 | C008 | Setúbal | 38.52 | -9.00 | A | A3 | L2 | E2 |
| pmar01 | C003 | Setúbal | 38.57 | -8.89 | A | A1 | L2 | E3 |
| pmar02 | C027 | Setúbal | 38.58 | -8.88 | A | A1 | L2 | E3 |
| pmtd01 | C032 | Setúbal | 38.55 | -8.89 | A | A1 | L2 | E3 |
| pmtd02 | C001 | Setúbal | 38.55 | -8.89 | A | A1 | L2 | E3 |
| pmqa01 | C032 | Setúbal | 38.56 | -8.94 | A | A1 | L2 | E3 |
| mnhf01 | C002 | Setúbal | 38.70 | -8.33 | C | C1 | L1 | E3 |
| mnhf02 | C012 | Setúbal | 38.70 | -8.33 | C | C1 | L1 | E3 |
| mnhf03 | C033 | Setúbal | 38.70 | -8.33 | C | C1 | L1 | E1 |
| mnhf04 | C030 | Setúbal | 38.70 | -8.33 | C | C1 | L1 | E3 |
| mnhf05 | C001 | Setúbal | 38.70 | -8.32 | C | C1 | L1 | E3 |
| mnhf06 | C001 | Setúbal | 38.70 | -8.32 | C | C1 | L1 | E3 |
| mnhf07 | C001 | Setúbal | 38.70 | -8.32 | C | C1 | L1 | E3 |
| mnhf08 | C003 | Setúbal | 38.70 | -8.32 | C | C1 | L1 | E3 |
| mnhf09 | C079 | Setúbal | 38.70 | -8.32 | C | C1 | L1 | E3 |
| mnhf10 | C008 | Setúbal | 38.70 | -8.32 | C | C1 | L1 | E3 |
| ezhg01 | C012 | Évora | 38.87 | -7.59 | A | A1 | L1 | E1 |
| ezhg02 | C034 | Évora | 38.87 | -7.59 | A | A1 | L1 | E1 |
| ezhg03 | C013 | Évora | 38.87 | -7.59 | A | A1 | L1 | E3 |
| ezhg04 | C010 | Évora | 38.87 | -7.59 | A | A1 | L1 | E1 |
| ezhg05 | C002 | Évora | 38.87 | -7.59 | A | A1 | L1 | E1 |
| ezhg06 | C002 | Évora | 38.87 | -7.59 | A | A1 | L1 | E3 |
| ezhg07 | C002 | Évora | 38.87 | -7.59 | A | A1 | L1 | E1 |
| ezhg08 | C002 | Évora | 38.87 | -7.59 | A | A1 | L1 | E3 |
| ezhg09 | C002 | Évora | 38.87 | -7.59 | A | A1 | L1 | E1 |
| ezhg10 | C080 | Évora | 38.87 | -7.59 | A | A1 | L1 | E1 |
| ezhg11 | C035 | Évora | 38.87 | -7.59 | A | A1 | L1 | E3 |
| ezhg12 | C008 | Évora | 38.87 | -7.59 | A | A1 | L1 | E1 |
| ezhg13 | C023 | Évora | 38.87 | -7.59 | A | A1 | L1 | E1 |
| ezhg14 | C006 | Évora | 38.87 | -7.59 | A | A1 | L1 | E3 |
| ezhg15 | C002 | Évora | 38.87 | -7.59 | A | A1 | L1 | E1 |
| ezhg16 | C023 | Évora | 38.87 | -7.59 | A | A1 | L1 | E3 |
| ezhg17 | C081 | Évora | 38.87 | -7.59 | A | A1 | L1 | E1 |
| ezhg19 | C002 | Évora | 38.87 | -7.59 | A | A1 | L1 | E1 |
| ezhg20 | C002 | Évora | 38.87 | -7.59 | A | A1 | L1 | E1 |
| evce02 | C036 | Évora | 38.60 | -7.89 | A | A1 | L2 | E3 |
| evce03 | C003 | Évora | 38.60 | -7.89 | A | A1 | L2 | E1 |
| evce04 | C036 | Évora | 38.60 | -7.89 | A | A1 | L2 | E1 |
| evce05 | C001 | Évora | 38.60 | -7.89 | A | A1 | L2 | E1 |
| evce06 | C002 | Évora | 38.60 | -7.89 | A | A1 | L2 | E3 |
| evce07 | C005 | Évora | 38.60 | -7.89 | A | A1 | L2 | E3 |
| evce08 | C002 | Évora | 38.60 | -7.89 | A | A1 | L2 | E1 |
| evce09 | C001 | Évora | 38.60 | -7.89 | A | A1 | L2 | E3 |
| evce10 | C001 | Évora | 38.60 | -7.89 | A | A1 | L2 | E1 |
| evce11 | C003 | Évora | 38.60 | -7.89 | A | A1 | L2 | E1 |
| evce12 | C001 | Évora | 38.60 | -7.89 | A | A1 | L2 | E3 |
| eveb01 | C001 | Évora | 38.58 | -7.97 | A | A2 | L1 | E1 |
| eveb03 | C002 | Évora | 38.58 | -7.97 | A | A2 | L1 | E1 |
| eveb04 | C015 | Évora | 38.57 | -7.97 | A | A2 | L1 | E1 |
| eveb05 | C002 | Évora | 38.58 | -7.97 | A | A2 | L1 | E1 |
| eveb06 | C001 | Évora | 38.58 | -7.97 | A | A2 | L1 | E3 |
| eveb07 | C006 | Évora | 38.58 | -7.97 | A | A2 | L1 | E1 |
| pvcp01 | C001 | Évora | 38.90 | -8.01 | A | A1 | L1 | E4 |
| pvcp02 | C082 | Évora | 38.89 | -8.01 | A | A1 | L1 | E1 |
| pvcp03 | C005 | Évora | 38.89 | -8.01 | A | A1 | L1 | E2 |
| pvcp04 | C034 | Évora | 38.90 | -8.01 | A | A1 | L1 | E3 |
| pvbr02 | C083 | Évora | 38.90 | -8.02 | A | A1 | L1 | E1 |
| pvbr04 | C001 | Évora | 38.90 | -8.02 | A | A1 | L1 | E1 |
| pvff01 | C002 | Évora | 38.90 | -8.02 | A | A1 | L2 | E3 |
| pvoa01 | C084 | Évora | 38.90 | -8.02 | A | A2 | L2 | E3 |
| evjp01 | C006 | Évora | 38.57 | -7.91 | A | A1 | L2 | E1 |
| atsl01 | C006 | Évora | 38.26 | -7.98 | A | A2 | L1 | E3 |
| atsl02 | C001 | Évora | 38.26 | -7.98 | A | A2 | L1 | E1 |
| atsl03 | C002 | Évora | 38.26 | -7.98 | A | A2 | L1 | E1 |
| atsl04 | C001 | Évora | 38.26 | -7.98 | A | A2 | L1 | E1 |
| atsl05 | C085 | Évora | 38.26 | -7.98 | A | A2 | L1 | E3 |
| atsl06 | C001 | Évora | 38.26 | -7.98 | A | A2 | L1 | E3 |
| atsl07 | C001 | Évora | 38.26 | -7.98 | A | A2 | L1 | E1 |
| atsl08 | C086 | Évora | 38.26 | -7.98 | A | A2 | L1 | E1 |
| atsl09 | C087 | Évora | 38.26 | -7.98 | A | A2 | L1 | E3 |
| atsl10 | C001 | Évora | 38.26 | -7. 978418 | A | A2 | L1 | E1 |
| atsl11 | C001 | Évora | 38.26 | -7.98 | A | A2 | L1 | E3 |
| atsl12 | C088 | Évora | 38.26 | -7.98 | A | A2 | L1 | E3 |
| atsl13 | C001 | Évora | 38.26 | -7.98 | A | A2 | L1 | E3 |
| atcp01 | C089 | Évora | 38.26 | -7.98 | A | A1 | L2 | E1 |
| atct01 | C090 | Évora | 38.26 | -7.99 | A | A2 | L2 | E1 |
| alcp01 | C001 | Évora | 38.58 | -7.36 | C | C1 | L1 | E1 |
| alcp02 | C001 | Évora | 38.58 | -7.36 | C | C1 | L1 | E1 |
| alcp03 | C091 | Évora | 38.58 | -7.36 | C | C1 | L1 | E1 |
| alcp05 | C001 | Évora | 38.58 | -7.36 | C | C1 | L1 | E1 |
| alcp06 | C010 | Évora | 38.58 | -7.36 | C | C1 | L1 | E3 |
| alcp07 | C001 | Évora | 38.58 | -7.36 | C | C1 | L1 | E1 |
| alcp08 | C001 | Évora | 38.58 | -7.36 | C | C1 | L1 | E1 |
| alcp09 | C001 | Évora | 38.58 | -7.35 | C | C1 | L1 | E1 |
| alcp10 | C001 | Évora | 38.58 | -7.35 | C | C1 | L1 | E1 |
| evjr01 | C001 | Évora | 38.68 | -7.77 | C | C1 | L1 | E1 |
| evjr02 | C001 | Évora | 38.68 | -7.77 | C | C1 | L1 | E1 |
| evjr03 | C001 | Évora | 38.68 | -7.77 | C | C1 | L1 | E1 |
| evjr04 | C001 | Évora | 38.68 | -7.77 | C | C1 | L1 | E1 |
| evjr05 | C004 | Évora | 38.68 | -7.77 | C | C1 | L1 | E3 |
| evjr06 | C001 | Évora | 38.68 | -7.78 | C | C1 | L1 | E1 |
| evjr07 | C001 | Évora | 38.68 | -7.78 | C | C1 | L1 | E2 |
| evjr08 | C002 | Évora | 38.68 | -7.78 | C | C1 | L1 | E1 |
| evjr09 | C001 | Évora | 38.68 | -7.78 | C | C1 | L1 | E1 |
| evjr10 | C092 | Évora | 38.68 | -7.78 | C | C1 | L1 | E1 |
| vdhg01 | C002 | Évora | 38.19 | -7.83 | C | C1 | L1 | E3 |
| vdhg02 | C001 | Évora | 38.19 | -7.83 | C | C1 | L1 | E1 |
| vdhg03 | C001 | Évora | 38.20 | -7.83 | C | C1 | L1 | E1 |
| vdhg04 | C002 | Évora | 38.20 | -7.83 | C | C1 | L1 | E3 |
| vdhg05 | C002 | Évora | 38.20 | -7.83 | C | C1 | L1 | E3 |
| vdhg06 | C001 | Évora | 38.20 | -7.83 | C | C1 | L1 | E1 |
| vdhg07 | C002 | Évora | 38.20 | -7.83 | C | C1 | L1 | E1 |
| vdhg08 | C001 | Évora | 38.20 | -7.83 | C | C1 | L1 | E1 |
| vdhg09 | C003 | Évora | 38.20 | -7.83 | C | C1 | L1 | E1 |
| vdhg10 | C001 | Évora | 38.20 | -7.83 | C | C1 | L1 | E1 |
| rdbq01 | C002 | Évora | 38.66 | -7.55 | C | C1 | L1 | E1 |
| rdbq02 | C010 | Évora | 38.66 | -7.55 | C | C1 | L1 | E1 |
| rdbq03 | C021 | Évora | 38.66 | -7.55 | C | C1 | L1 | E1 |
| rdbq04 | C002 | Évora | 38.66 | -7.55 | C | C1 | L1 | E3 |
| rdbq05 | C010 | Évora | 38.66 | -7.55 | C | C1 | L1 | E3 |
| rdbq06 | C002 | Évora | 38.66 | -7.55 | C | C1 | L1 | E1 |
| rdbq07 | C013 | Évora | 38.66 | -7.55 | C | C1 | L1 | E2 |
| rdbq09 | C002 | Évora | 38.66 | -7.55 | C | C1 | L1 | E1 |
| rdbq10 | C002 | Évora | 38.66 | -7.55 | C | C1 | L1 | E1 |
| mopb01 | C001 | Beja | 38.13 | -7.40 | C | C1 | L1 | E1 |
| mopb02 | C001 | Beja | 38.13 | -7.40 | C | C1 | L1 | E1 |
| mopb04 | C001 | Beja | 38.12 | -7.41 | C | C1 | L1 | E3 |
| mopb05 | C001 | Beja | 38.12 | -7.41 | C | C1 | L1 | E3 |
| mopb06 | C001 | Beja | 38.12 | -7.41 | C | C1 | L1 | E1 |
| mopb07 | C014 | Beja | 38.12 | -7.41 | C | C1 | L1 | E3 |
| mopb08 | C001 | Beja | 38.12 | -7.40 | C | C1 | L1 | E1 |
| mopb09 | C001 | Beja | 38.12 | -7.40 | C | C1 | L1 | E3 |
| mopb10 | C001 | Beja | 38.13 | -7.40 | C | C1 | L1 | E3 |
| sphm01 | C001 | Beja | 38.12 | -7.56 | C | C1 | L1 | E1 |
| sphm03 | C002 | Beja | 38.12 | -7.56 | C | C1 | L1 | E3 |
| sphm04 | C001 | Beja | 38.12 | -7.56 | C | C1 | L1 | E3 |
| sphm05 | C001 | Beja | 38.12 | -7.56 | C | C1 | L1 | E3 |
| sphm07 | C010 | Beja | 38.12 | -7.56 | C | C1 | L1 | E1 |
| sphm08 | C001 | Beja | 38.12 | -7.56 | C | C1 | L1 | E3 |
| sphm10 | C093 | Beja | 38.12 | -7.55 | C | C1 | L1 | E3 |
| spql01 | C005 | Beja | 37.94 | -7.66 | C | C1 | L1 | E1 |
| spql03 | C001 | Beja | 37.94 | -7.66 | C | C1 | L1 | E1 |
| spql04 | C001 | Beja | 37.94 | -7.66 | C | C1 | L1 | E1 |
| spql05 | C001 | Beja | 37.94 | -7.66 | C | C1 | L1 | E1 |
| spql06 | C005 | Beja | 37.94 | -7.66 | C | C1 | L1 | E1 |
| spql07 | C094 | Beja | 37.94 | -7.66 | C | C1 | L1 | E1 |
| spql08 | C005 | Beja | 37.94 | -7.66 | C | C1 | L1 | E1 |
| spql09 | C095 | Beja | 37.94 | -7.66 | C | C1 | L1 | E1 |
| spql10 | C005 | Beja | 37.94 | -7.66 | C | C1 | L1 | E3 |

**A** – age category: ancient

**C** – age category: centenary

**A1** – age group: 400-599 years old

**A2** – age group: 600-999 years old

**A3** – age group: more than 1000 years old

**C1** – age group: 80-100 years old

**L1**– location: orchard

**L2** – location: isolated site

**E1** – endocarp profile 1: rounded base shape (position A) and medium weight (0.30–0.45 g).

**E2** – endocarp profile 2: rounded base shape (position A) and low weight (< 0.30 g)

**E3** – endocarp profile 3: acute base shape (position A) and medium weight (0.30–0.45 g).

**E4** – endocarp profile 4: acute base shape (position A) and low weight (< 0.30 g)

**Supplementary Table 2.** Agroecological information of the ten geographical districts under study (data collected in the first year of the prospection).

| **District** | **Temperature**  **mean(ºC)** | **Temperature**  **max(ºC)** | **Temperature**  **min(ºC)** | **Rainfall**  **(mm/year)** |
| --- | --- | --- | --- | --- |
| **Viseu** | 14.54 | 33.30 | 1.90 | 1617.80 |
| **Coimbra** | 16.41 | 32.6 | 3.60 | 1099.60 |
| **Castelo Branco** | 16.88 | 36.60 | 4.20 | 796.60 |
| **Leiria** | 15.47 | 29.80 | 3.80 | 1063.60 |
| **Portalegre** | 16.57 | 35.20 | 3.70 | 1038.00 |
| **Santarém** | 17.80 | 35.40 | 5.80 | 700.30 |
| **Lisboa** | 17.75 | 31.90 | 8.30 | 729.10 |
| **Setúbal** | 17.36 | 33.80 | 4.90 | 813.70 |
| **Évora** | 17.22 | 37.50 | 4.30 | 497.10 |
| **Beja** | 17.66 | 36.80 | 3.60 | 668.70 |
